# Spatial detection and consequences of nonrenal calcitriol production as assessed by targeted mass spectrometry imaging

**DOI:** 10.1172/jci.insight.181763

**Published:** 2024-06-25

**Authors:** Mark B. Meyer, Seong Min Lee, Shannon R. Cichanski, Diego F. Cobice, J. Wesley Pike

**Affiliations:** 1 Department of Nutritional Sciences, University of Wisconsin-Madison, Madison, Wisconsin, USA.; 2 Mass Spectrometry Centre, Biomedical Sciences Research Institute (BMSRI), School of Biomedical Sciences, Ulster University, Coleraine, Northern Ireland, United Kingdom.; 3 Department of Biochemistry, University of Wisconsin-Madison, Madison, Wisconsin, USA.

**Keywords:** Endocrinology, Calcium signaling, Cytokines, Macrophages

## Abstract

The immune benefits of vitamin D_3_ supplementation beyond calcium and phosphate maintenance are highly clinically debated. Kidney expression of CYP27B1 is the source of endocrine, circulating 1,25(OH)_2_D_3_ (active form of vitamin D) that maintains serum calcium and phosphate. 1,25(OH)_2_D_3_ may also be made by the CYP27B1 enzyme in nonrenal cells, like immune cells, in a process driven by cellular availability of 25(OH)D_3_ and inflammation. Due to the endocrine nature of 1,25(OH)_2_D_3_ in circulation, it is difficult to discern between these 2 sources. We recently created a regulatory deletion model of *Cyp27b1* (M1/M21-DIKO) where mice have normal inflammatory-regulated *Cyp27b1* expression in nonrenal tissues (unlike global *Cyp27b1*-KO) but no expression within the kidney. Here, utilizing on-tissue chemical derivatization and matrix assisted laser desorption ionization-mass spectrometry imaging (MALDI-MSI), we investigated the distribution of 1,25(OH)_2_D_3_ and 25(OH)D_3_ in the kidney, liver, spleen, and thymus. MALDI-MSI demonstrated increased 1,25(OH)_2_D_3_ in nonrenal tissues such as the spleen after vitamin D_3_ supplementation in M1/M21-DIKO mice. Additionally, from this, we found increased *Il4* and decreased *Tnfa* in the spleen after vitamin D_3_ supplementation. Taken together, these data demonstrate nonrenal production of 1,25(OH)_2_D_3_ in vivo and provide a consequence of vitamin D_3_ supplementation and nonrenal 1,25(OH)_2_D_3_ production in cytokine changes.

## Abstract 2

Mass spectrometry imaging in vivo reveals that nonrenal vitamin D hormone production leads to an antiinflammatory program after vitamin D supplementation.

## Introduction

Vitamin D has profound abilities to regulate many different biological processes; it can influence the differentiation of skeletal cells, change immune programming, process bile acids, control mineral channels, fight cancer cell proliferation, and promote antiinflammatory responses (1–4). The amount of vitamin D_3_ supplementation needed for these various tasks, however, has been widely debated as human clinical trials have failed to confirm the effects observed from cell culture and animal studies (5–7). These studies are all complicated by renal production of 1α,25(OH)_2_D_3_ (1,25[OH]_2_D_3_ calcitriol) (8). The ability of the body to regulate calcium (Ca) and phosphate (P) relies on proper functioning of vitamin D metabolism with CYP27B1 making 1,25(OH)_2_D_3_ and CYP24A1 degrading 1,25(OH)_2_D_3_. *CYP27B1* expression in the kidney is a highly regulated, endocrine hormone–controlled process with parathyroid hormone (PTH) inducing CYP27B1 and fibroblast growth factor 23 (FGF23) and 1,25(OH)_2_D_3_ suppressing CYP27B1 (8). *CYP24A1* expression is reciprocally regulated to *CYP27B1* in that PTH suppresses CYP24A1 and FGF23 and 1,25(OH)_2_D_3_ induces CYP24A1 (9, 10). The balance of these enzymes in the kidney controls vitamin D metabolism in the body.

Outside of kidney, regulation of *CYP27B1* and *CYP24A1* are quite different. *CYP27B1* expression is not controlled by the endocrine hormones PTH, FGF23, or 1,25(OH)_2_D_3_ (10). *CYP24A1* retains its ability to be regulated by 1,25(OH)_2_D_3_; however, FGF23 and PTH fail to change the expression in nonrenal tissues (9). Additionally, the nonrenal expression levels of *Cyp27b1* and *Cyp24a1* are 100- to 1,000-fold lower than what is observed in the kidney in mice (9, 10). However, these nonrenal cells do retain low and measurable expression of *Cyp27b1*. *Cyp27b1* in these tissues is induced by inflammatory signals like LPS, IL-1β, and others (11). It has also been demonstrated that macrophages can produce 1,25(OH)_2_D_3_ during sarcoidosis in humans (12). Substrate availability (25[OH]D_3_) for CYP27B1 is key to the physiologic regulation of vitamin D metabolism, as 25(OH)D_3_ bound to an albumin binding protein (vitamin D binding protein [DBP]) is transported through the cell surface receptors megalin and cubulin (13–15). These receptors have high expression in the kidney, so the availability of 25(OH)D_3_ remains high; however, other tissues have far lower expression and, thus, it is believed they may have lower availability of 25(OH)D_3_ (16). Additionally, a recent article examining 1,25(OH)_2_D_3_ levels in anephric patients found that low levels of circulating 1,25(OH)_2_D_3_ were present and this was postulated to be coming from nonrenal sources driven by substrate availability (17).

We recently solved the renal regulatory mechanism for *Cyp27b1* expression through key upstream enhancers in the proximal convoluted tubule cells (18) in intronic regions of the neighboring *Mettl1* and *Mettl121b* (*Eef1akmt3*) genes (10, 11). We used CRISPR-Cas9 editing to create a mouse (M1/M21–double intronic KO [M1/M21-DIKO]) with a similar phenotype to the global *Cyp27b1*-KO mouse with low serum Ca and P, low FGF23, very high levels of PTH, and many skeletal and mineral defects (11). The M1/M21-DIKO mouse had minimal expression of the *Cyp27b1* gene in the kidney and was resistant to endocrine hormone control by PTH, FGF23, or 1,25(OH)_2_D_3_. This animal also had altered vitamin D metabolism with low circulating 1,25(OH)_2_D_3_ and elevated levels of 25(OH)D_3_ among other metabolite changes (11). Critically, the M1/M21-DIKO mouse retained its basal expression and inflammatory induction of *Cyp27b1* in nonrenal tissues, including macrophages and T cells (11). We described this mouse as a kidney-specific, endocrine-deficient *Cyp27b1* pseudo-null mouse since the effects were confined to the kidney (11). We also demonstrated that, like the global *Cyp27b1*-KO mouse, the M1/M21-DIKO mouse could have its skeleton and mineral defects rescued by feeding a diet high in Ca, P, and lactose (2%, 1.25%, 20% lactose) for 12–16 weeks (11). This reduced the 1,25(OH)_2_D_3_ circulating levels below the lower limits of detection (LLOD) in the mouse. Therefore, we believe the dietary rescued M1/M21-DIKO mouse represents the ideal model with which to study vitamin D_3_ supplementation and the potential to isolate and demonstrate nonrenal production of 1,25(OH)_2_D_3_.

Routine and clinical measurements of both 25(OH)D_3_ and 1,25(OH)D_3_ in the serum or plasma are typically made with ELISA kits or a radioimmunoassay (RIA) assay (19); however, these methods lack detailed analysis of the multitude of vitamin D metabolite derivatives and can often be confounded by them (20, 21). Methods of liquid chromatography followed by tandem mass spectrometry (LC-MS/MS) greatly increases the ability to see these derivatives in the serum or plasma with absolute quantitation (22). Detection of tissue levels of vitamin D metabolites is not trivial, given the high lipid and cholesterol content of tissues. Furthermore, detecting the potential for production of those metabolites in tissues, as we propose here, is also complicated by the circulating 1,25(OH)_2_D_3_ production from the kidney. Recently, methods were developed for the targeted acquisition of spatial identification and relative quantitation of vitamin D metabolites through advances in MS imaging (MSI) (23). Serum detection of vitamin D metabolites by LC-MS/MS involves a chemical derivatization (CD) step to improve the ionization efficiency of steroidal compounds (20, 22). By taking this a step further, the derivatization can be accomplished on tissue sections (on-tissue CD [OTCD]) to enable tissue detection of vitamin D metabolites. Additionally, spatial information within the tissue can be obtained when linked to matrix assisted laser desorption ionization (MALDI) followed by MS detection (23). Thus far, this OTCD-MALDI-MSI pipeline of analysis has been completed to identify both 25(OH)D_3_ and 1,25(OH)_2_D_3_ in the mouse kidney of WT mice in proof-of-principle experiments (23).

In this current study, we combined this latest technology of MSI utilizing OTCD-MALDI-MSI to identify and quantify the tissue-level production of 1,25(OH)_2_D_3_ and 25(OH)D_3_. With our M1/M21-DIKO mouse, we have eliminated the circulating 1,25(OH)_2_D_3_ and focus on the detection of 1,25(OH)_2_D_3_ resident in the tissues themselves. Using this unique MSI technique, we demonstrate detection and spatial location of 1,25(OH)_2_D_3_ and 25(OH)D_3_ in tissues outside of the kidney. We hypothesize that vitamin D_3_ supplementation will increase 25(OH)D_3_ circulation and tissue availability, as well as the production of 1,25(OH)_2_D_3_ in tissues outside of the kidney in the M1/M21-DIKO mouse. This, in turn, may prove beneficial for inflammatory disease amelioration and provide the missing link between cell and human studies. In these studies, we have demonstrated nonrenal detection and production of 1,25(OH)_2_D_3_ in vivo, as well as gene expression consequences, in response to vitamin D_3_ supplementation.

## Results

### MSI validation of vitamin D metabolites in WT and Cyp27b1-KO mice.

**Figure 1:**
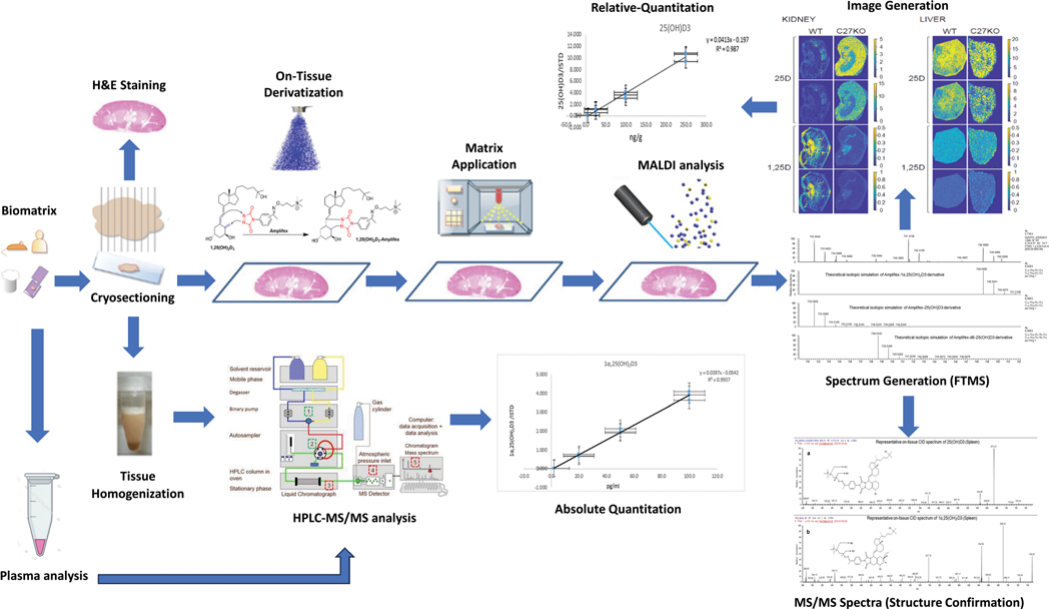
Mass spectrometry imaging workflow overview of tissue analysis. Isolated tissues are sectioned, and tissues samples are split for H&E staining, tissue homogenization (for absolute quantitation), and OTCD-MALDI-MSI for spatial distribution assessment and relative quantitation. Samples for OTCD-MALDI-MSI analysis undergo reagent application (Ampiflex) with the inclusion of a stable isotope for relative quantitation and subsequent MALDI matrix application for MALDI-MSI analysis. Protonated ions for targeted metabolites are selected for image generation and relative quantitation with further ion isolation for structural confirmatory analysis (MS/MS) The plasma is taken for systemic metabolite contribution and analyzed by HPLC-MS/MS along with tissue homogenate for absolute quantitation of vitamin D metabolites.

We used a similar workflow and procedure as previously reported for MS interrogation of targeted metabolite imaging and plasma metabolites from tissues (23) (Figure 1). The mice lacking mature, full-length CYP27B1 protein (*Cyp27b1*-KO) are known to have elevated levels of substrate 25(OH)D_3_ compared with their WT littermates (10, 11). We confirmed these elevated plasma levels of 25(OH)D_3_ and confirmed that 1,25(OH)_2_D_3_ is absent compared with WT mice, in this study. In Supplemental Figure 1A (supplemental material available online with this article; https://doi.org/10.1172/jci.insight.181763DS1), mice (male and female) were examined by LC-MS/MS for both 25(OH)D_3_ and 1,25(OH)_2_D_3_ concentrations in the plasma. WT mice had an average plasma 25(OH)D_3_ concentration of 15.4 ± 1.1 ng/mL and a 1,25(OH)_2_D_3_ concentration of 23.7 ± 1.9 pg/mL (Supplemental Figure 1), consistent with our prior studies (10, 11). The *Cyp27b1*-KO (C27-KO) mice had a 25(OH)D_3_ concentration of 77.9 ± 2.8 ng/mL (Supplemental Figure 1) and 1,25(OH)_2_D_3_ that was found in the linear range but fell below the lower limit of quantitation (<LLOQ); however, these values were above the LLOD and are, therefore, included and displayed in red. Plasma LLOQ for 25(OH)D_3_ is ≤ 2 ng/mL and for 1,25(OH)_2_D_3_ is ≤ 5 pg/mL. Both the 25(OH)D_3_ and 1,25(OH)_2_D_3_ *Cyp27b1*-KO versus WT were statistically significant with *P* < 0.001 in each case by *t* test (1 tailed) analysis. Both male and female mice were included in the replicates; no differences were found, and therefore, the results are reported as mixed. These mice confirm our previously measured plasma 25(OH)D_3_ and 1,25(OH)_2_D_3_ concentrations for both the WT and *Cyp27b1*-KO mice (10, 11).

**Figure 2:**
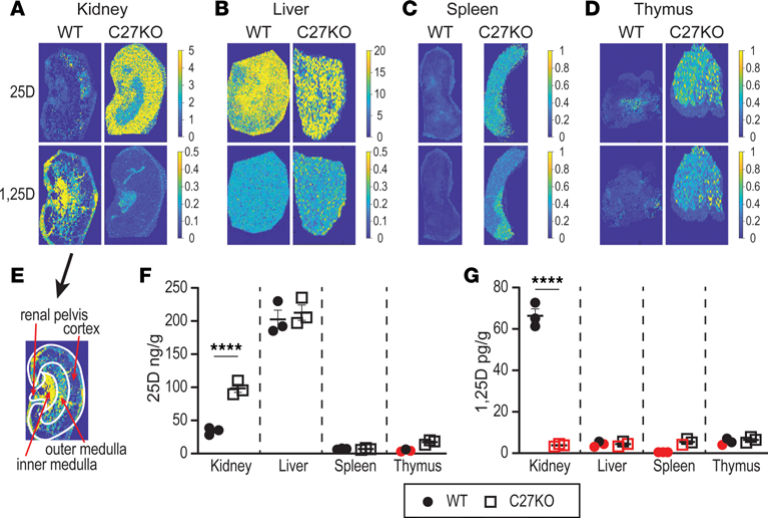
Tissue localization and relative quantitation of 25(OH)D_3_ and 1,25(OH)_2_D_3_ in *Cyp27b1*-KO and WT mice. (**A**–**D**) MALDI-MSI was performed on both *Cyp27b1*-KO and WT littermates in the kidney (**A**), liver (**B**), spleen (**C**), and thymus (**D**). Scale for each metabolite shown to the right of each tissue image (additional images in Supplemental data). Intensity was normalized by stable isotope internal standard protonated mass. Signal intensity is depicted by parula color scale for semiquantitative assessment on the scale shown. (**E**) Schematic of kidney anatomy overlaid with panel from 1,25D WT from **A**. (**F** and **G**) The relative quantitation of biological triplicates is shown for the 25(OH)D_3_ (**F**, ng/g) and 1,25(OH)_2_D_3_ (**G**, pg/g) for each tissue (all values in Supplemental Figure 1). Data points that fall below the lower limits of quantitation (<LLOQ) but above the lower limits of detection (LLOD) are shown in red. *n* = 3 for all tissues. Unpaired *t* tests (1 tailed) were performed. ****P < 0.0001, C27-KO versus WT.

We then investigated tissue MALDI-MSI for both WT and *Cyp27b1*-KO animals. Both WT and *Cyp27b1*-KO animals were fed a chow diet (3.4 IU/g of vitamin D) until 8 weeks of age. Mice were then perfused with PBS, and tissues were collected. Animal body weights, bone mineral density (BMD), and serum Ca, P, PTH, and in-tact FGF23 (iFGF23) are reported in Supplemental Figure 3. Importantly, the intratissue systemic contribution of blood was not detected in the tissues, indicating that the perfusion was complete. MALDI-MSI was performed for kidney, liver, spleen, and thymus, as seen in Figure 2. Representative MSIs are shown in Figure 2, A–D, with 25(OH)D_3_ (top) and 1,25(OH)_2_D_3_ (bottom) of each panel. Relative quantification from the raw MSI data for each panel and biological replicate sample are displayed in Figure 2, F and G. Representative images from all biological triplicates and the full table of metabolites are shown in Supplemental Figures 1 and 2, respectively. The current resolution of MSI is based on the laser used for tissue ablation, which is near 35 μm in size, thus ablating approximately 20–25 cells at a time. As can be observed in Figure 2A (upper), the WT kidney shows small amounts of 25(OH)D_3_ present, indicated by the low ion relative intensity or abundance compared with areas of high ion relative intensity and abundance. The WT kidney 1,25(OH)_2_D_3_ (Figure 2A) shows a high relative intensity of the 1,25(OH)_2_D_3_ metabolite. In contrast to the WT metabolite levels of the 25(OH)D_3_, the *Cyp27b1*-KO mouse (C27-KO) has high levels of 25(OH)D_3_. As expected, the *Cyp27b1*-KO mouse is incapable of making 1,25(OH)_2_D_3_ and, therefore, the MSIs show very low levels of ion intensity and metabolite detection. From an overlay of a schematic of the mouse kidney anatomy (Figure 2E), we found that 1,25(OH)_2_D_3_ appeared in both the cortex and medulla, with the inner medulla having a much higher relative ion intensity than the cortex or outer medulla. Quantification of these data as shown in Figure 2F measure the 25(OH)D_3_ of the WT kidney to be 34.1 ± 3.1 ng/g and the *Cyp27b1*-KO 98.5 ± 6.1 ng/g. The 1,25(OH)_2_D_3_ quantification (Figure 2G) of WT kidney was 66.4 ± 3.4 pg/g and *Cyp27b1*-KO was 3.8 ± 0.4 pg/g (below the LLOQ). These data validate the method against the prior publication for MSI of vitamin D metabolites (23) and are in line with those measured for the plasma values (Supplemental Table 1) and from our prior work (10, 11).

The liver is the source of 25(OH)D_3_ production by the CYP2R1 enzyme and, thus, is expected to have the highest tissue levels of 25(OH)D_3_ in the mouse. In Figure 2B, MSI confirms this expectation, as the levels of 25(OH)D_3_ were high in both the WT and *Cyp27b1*-KO mice. Quantification of these data in Figure 2F show there was no statistical difference in the levels of 25(OH)D_3_ between the WT and *Cyp27b1*-KO mice (202.5 ± 13.9 and 212.7 ± 11.6 ng/g, respectively; Figure 2F). The levels of 1,25(OH)_2_D_3_ (Figure 2G) in the liver were low with 2 of the 3 replicates falling below the LLOQ for both WT (4.4 ± 0.7 pg/g) and *Cyp27b1*-KO (4.5 ± 0.7 pg/g; Figure 2G). Both spleen and thymus contained very little 25(OH)D_3_ for either the WT or *Cyp27b1*-KO mice (spleen, 7.1 ± 0.5 and 7.0 ± 0.8 ng/g; thymus, 4.6 ± 0.9 and 17.3 ± 2.0 ng/g; Figure 2F). While the *Cyp27b1*-KO appeared to have more 25(OH)D_3_ in the thymus compared with the WT, this was not a statistically significant increase. The levels of 1,25(OH)_2_D_3_ in the spleen for the WT mouse was below the LLOD (<2 pg/g), and the *Cyp27b1*-KO was at the LLOQ (Figure 2G). Similarly, the 1,25(OH)_2_D_3_ levels in the thymus for either mouse were also at or below the LLOQ. Taken together, data from the WT and *Cyp27b1*-KO mice confirm our expectations and/or previous data (23) that the WT kidney should contain the highest levels of 1,25(OH)_2_D_3_, the liver should be a reservoir for 25(OH)D_3_, *Cyp27b1*-KO mice should have elevated 25(OH)D_3_ in the kidney, and the remaining tissues should have very little to no detectable 1,25(OH)_2_D_3_.

### Vitamin D metabolite profile of the M1/M21-DIKO mouse.

**Figure 3:**
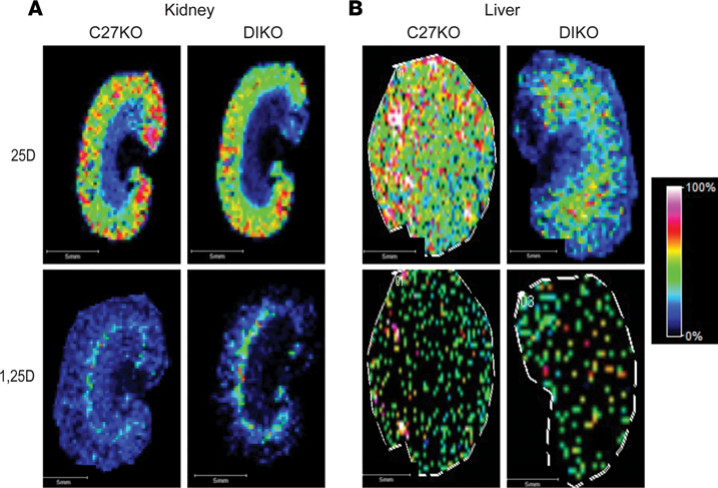
Qualitative Comparison of *Cyp27b1*-KO and M1/M21-DIKO mice by MALDI-MSI. (**A** and **B**) MALDI-MSI was performed and the representative images from the kidney (**A**) and liver (**B**) for both the *Cyp27b1*-KO (C27-KO) and M1/M21-DIKO (DIKO) mice, and 25(OH)D_3_ (25D) and 1,25(OH)_2_D_3_ (1,25D) were measured and displayed. The intensity scale used was based on total ion count (TIC) normalization using rainbow color scale, from black (0%) to white (100%) ion intensity.

Next, we defined the tissue metabolite levels of our experimental M1/M21-DIKO mice. These mice have a genomic deletion in enhancers that render the *Cyp27b1* gene incapable of responding to any hormonal signals from FGF23, PTH, or 1,25(OH)_2_D_3_ as well as a reduced basal expression of *Cyp27b1* (11). As mentioned, the important feature of the M1/M21-DIKO mouse is that any nonrenal, inflammatory-induced response of *Cyp27b1* is unchanged from WT expression. Therefore, we hypothesize that the M1/M21-DIKO mouse is an ideal model with which to test the nonrenal production of 1,25(OH)_2_D_3_ in inflammation and inflammatory disease progression with the absence of renal produced 1,25(OH)_2_D_3_. Since the plasma levels of vitamin D metabolites mirror those of the *Cyp27b1*-KO mouse, we expected the tissue levels of vitamin D metabolites in the M1/M21-DIKO mouse to also mirror the MSI of the *Cyp27b1*-KO mouse. In Figure 3, we assessed the qualitative MSI profile of the M1/M21-DIKO versus the *Cyp27b1*-KO, with both mice on chow diet (3.4 IU/g vitamin D). Here, unlike Figure 2, qualitative MS images were reconstructed using Fleximaging in contrast to the semiquantitative evaluation performed using MSiReader software. The intensity scale was based on total ion count using the rainbow color scale rather than the internal standard peak normalization (parula color scale) used for semiquantitative assessment. Given this difference, these values are qualitative and not semiquantitative. We found that, in both the kidney and the liver (Figure 3, A and B), the M1/M21-DIKO (DIKO) mouse was comparable to the *Cyp27b1*-KO (C27-KO) mouse. The 25(OH)D_3_ levels were high in the kidney tissue, and the 1,25(OH)_2_D_3_ levels were low in the M1/M21-DIKO mouse. We previously found that M1/M21-DIKO mice on a chow diet did have low but detectable levels of 1,25(OH)_2_D_3_ in the plasma compared with *Cyp27b1*-KO (11). This appears to be replicated in the kidney MSI with slightly elevated levels of 1,25(OH)_2_D_3_ as evidenced by increased ion intensity compared with the *Cyp27b1*-KO; however, the relative quantitation was not performed. The liver contained elevated levels of 25(OH)D_3_ and no 1,25(OH)_2_D_3_ metabolites.

### Effect of vitamin D_3_ supplementation on tissue metabolite levels in the M1/M21-DIKO mouse.

Since the M1/M21-DIKO mouse lacks kidney *Cyp27b1* expression and regulation, we can exploit these features to associate vitamin D_3_ supplementation of varying levels of vitamin D diets with health and disease outcomes. Therefore, we examined the tissue levels of both 25(OH)D_3_ and 1,25(OH)_2_D_3_ after vitamin D_3_ supplementation. However, before supplementation, the mineral deficiencies of the M1/M21-DIKO mouse need to be rescued to recover and maintain skeletal density and growth. Through dietary rescue with a high Ca, high P, lactose-containing diet, the M1/M21-DIKO mice grow to similar size and similar skeletal density to age-matched WT mice (11). We found that this dietary rescue requires a minimum of 12 weeks of rescue diet (2% Ca, 1.25% P, 20% lactose) to recover serum Ca, P, and iFGF23 including lowering the serum PTH to WT levels (Supplemental Figure 3), and all values replicated our recent study (11). Therefore, to establish levels of vitamin D metabolites in the tissues, we first rescued mice for 12 weeks using the standard rescue diet (2% Ca, 1.25% P, 20% lactose containing 1.5 IU/g of vitamin D) and then switched to a diet containing either 0 IU/g of vitamin D (vitamin D–deficient diet) or diet containing 20 IU/g of vitamin D daily (high-D diet) for another 4 weeks, resulting in a total experimental timeline of 16 weeks. We examined the extremes (0 IU and 20 IU) by MSI to capture a maximal potential effect (24). This high dietary supplementation at 20 IU was previously shown to elevate the serum levels and tissue levels of 25(OH)_2_D_3_, which was our desired outcome (24). We also examined a 5 IU vitamin D_3_ supplementation as a moderate dose; however, given technical constraints, these mice were not examined by MSI in this study and will be examined in a future study. The mice after the additional 4 weeks of 0 IU or 20 IU diet were unchanged outside of femoral BMD in the female mice (Supplemental Figure 3).

**Figure 4:**
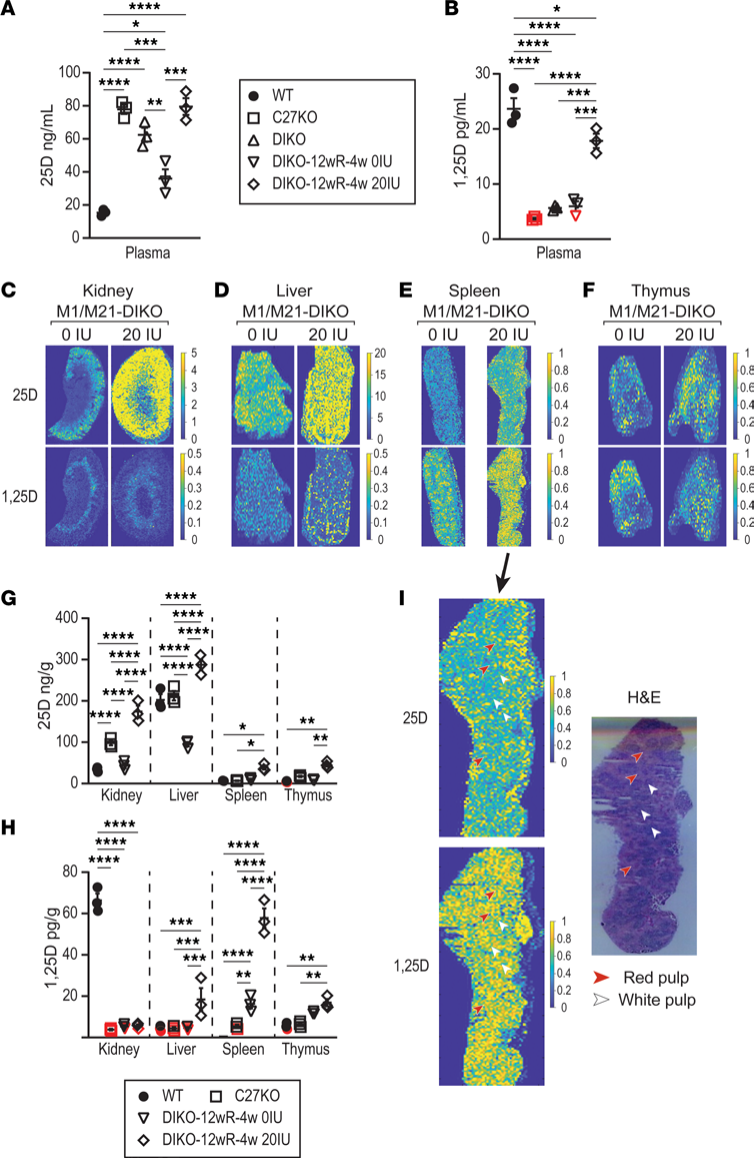
Detection of nonrenal 1,25(OH)_2_D_3_ production after vitamin D_3_ supplementation. (**A** and **B**) Plasma levels of 25(OH)D_3_ (**A**, 25D, ng/mL) and 1,25(OH)_2_D_3_ (**B**, 1,25D, pg/mL) as detected by LC-MS/MS in the WT littermates, *Cyp27b1*-KO (C27-KO), M1/M21-DIKO (DIKO), M1/M21-DIKO 12-week rescue diet followed by 4 weeks of 0 IU vitamin D diet (12wR-4w 0 IU), and M1/M21-DIKO 12-week rescue diet followed by 4 weeks of 20 IU vitamin D diet (12wR-4w 20 IU). One-way ANOVA with multiple comparison Tukey post hoc test. *P < 0.05; **P < 0.01; ***P < 0.001; ****P < 0.0001. (**C**–**F**) MSI was performed and displayed for kidney (**C**), liver (**D**), spleen (**E**), and thymus (**F**) in the M1/M21-DIKO 12wR-4w 0 IU and M1/M21-DIKO 12wR-4w 20 IU. Signal intensity is depicted by parula color scale for semiquantitative assessment on the scale shown (additional images in Supplemental Figure 2). (**G** and **H**) The relative quantitation of biological triplicates is shown for the 25(OH)D_3_ (**G**, ng/g) and 1,25(OH)_2_D_3_ (**H**, pg/g) for each tissue (all values in Supplemental Figure 1). Data points that fall below the lower limits of quantitation (<LLOQ) but above the LLOD are shown in red. *n* = 3 for all tissues. One-way ANOVA (genotypes within tissues) with multiple comparison Tukey post hoc test. *P < 0.05; **P < 0.01; ***P < 0.001; ****P < 0.0001. (**I**) H&E staining of thymus section with red and white pulp indicated. Overlaid data from spleen 25D and 1,25D panels from **E**.

In Figure 4, A and B, we examined the plasma levels of 25(OH)D_3_ and 1,25(OH)_2_D_3_ by LC-MS/MS. The WT and *Cyp27b1*-KO mice (chow diet) were compared with the M1/M21-DIKO (DIKO; chow diet) mice as well as the M1/M21-DIKO mice with a 12-week rescue diet followed by 4 weeks of either 0 or 20 IU vitamin D diet (DIKO-12wR-4w 0IU and DIKO-12wR-4w 20IU, respectively) with the full values included in Supplemental Figure 1. It is important to note that these mice were on different diets, given the goals of this study: to remove circulating 1,25(OH)_2_D_3_ in the M1/M21-DIKO mice and to test vitamin D_3_ supplementation. Rescue diets and vitamin D_3_ supplementation diets for WT and *Cyp27b1*-KO mice can cause different serum vitamin D metabolite profiles (24) and will be tested in the future. In Figure 4A, we see the M1/M21-DIKO mice on the chow diet showed a similar (not significantly different) 25(OH)D_3_ profile compared with the *Cyp27b1*-KO mouse (DIKO, 62.5 ± 4.2 ng/mL; C27-KO, 77.9 ± 2.8 ng/mL; Figure 4A). The 0 IU vitamin D diet did reduce the plasma 25(OH)D_3_ concentration in the DIKO mice (62.5 ± 4.2 ng/mL to 36.0 ± 5.7 ng/mL; Figure 4A), and the increase of vitamin D to 20 IU raised the 25(OH)D_3_ concentration to 79.6 ± 5.1 ng/mL (Figure 4A). The M1/M21-DIKO mouse was on a normal chow diet (unrescued; mouse phenotype: high PTH, low Ca, P, and FGF23) for 8 weeks prior to the harvest for metabolites, and these serum metabolites match our previous study (11). The M1/M21-DIKO mice were fed 12-week rescue followed by 4 weeks of a 0 IU or 20 IU diet equaling 16 weeks of total diet after wean. The 1,25(OH)_2_D_3_ levels in Figure 4B show the normal WT levels of 23.7 ± 1.9 pg/mL were greatly reduced in the *Cyp27b1*-KO, M1/M21-DIKO, and the M1/M21-DIKO with the 0 IU diet, many of these data points were at or below the LLOQ but above the LLOD (3.7 ± 0.2, 5.7 ± 0.3, and 6.0 ± 0.9 pg/mL, respectively; Figure 4B). Surprisingly, the 1,25(OH)_2_D_3_ concentration in the M1/M21-DIKO mouse with the 20 IU diet was significantly increased to 17.8 ± 1.3 pg/mL (Figure 4B), which was approaching WT levels (~23 pg/mL) though still significantly reduced (*P* < 0.05). These increased 1,25(OH)_2_D_3_ levels could be coming from an increased production in the kidney or perhaps from nonrenal tissues; therefore, we turned to MSI in the kidney, liver, spleen, and thymus to examine this possibility.

The MSI data for the kidney, liver, spleen, and thymus are shown in Figure 4, C–F, with the quantification listed in Figure 4, G and H. A complete table of the quantification and remaining representative images are shown in Supplemental Figures 1 and 2. The first and most obvious location for the increased 1,25(OH)_2_D_3_ in the 20 IU diet could be from the kidney. As seen in Figure 4C, reduction of vitamin D in the diet (0 IU) and reduction of vitamin D in the plasma (Figure 4A) leads to a reduced amount of 25(OH)D_3_ in the kidney tissue in comparison with the 20 IU diet, as evidenced by the increased ion relative intensity. The quantification of the increase is detailed in Figure 4G, where the 25(OH)D_3_ of the M1/M21-DIKO mouse with 0 IU diet is unchanged from the WT mice. However, 25(OH)D_3_ of the M1/M21-DIKO mouse with the 20 IU diet is significantly increased compared with the *Cyp27b1*-KO mouse (C27-KO, 98.5 ± 6.1 ng/g; DIKO 20 IU, 174.0 ± 14.4 ng/g; Figure 4G). Despite this increase in the 25(OH)D_3_ levels, it did not appear to cause any increased 1,25(OH)_2_D_3_ production in the kidney of the M1/M21-DIKO mouse on the 20 IU diet. The MSIs (Figure 4C) show little to no presence of 1,25(OH)_2_D_3_, and the quantification in Figure 4H demonstrates that the M1/M21-DIKO mice with either 0 IU or 20 IU diet were below the LLOQ yet above the LLOD, equivalent to the *Cyp27b1*-KO mice (C27-KO, 3.8 ± 0.4 pg/g; DIKO 0 IU, 5.3 ± 0.6 pg/g; DIKO 20 IU, 5.6 ± 0.7 pg/g; Figure 4H). It would appear from these data that the source of the increased 1,25(OH)_2_D_3_ in the plasma is not derived from the kidney.

The reservoir of 25(OH)D_3_ in the liver of the WT and *Cyp27b1*-KO mice was reduced in the M1/M21-DIKO mice on the 0 IU diet (WT, 202.5 ± 13.9 ng/g; C27-KO, 212.7 ± 11.6 ng/g; DIKO 0 IU, 95.3 ± 5.3 ng/g; Figure 4G) and was markedly increased in the M1/M21-DIKO mice on the 20 IU diet (287.5 ± 13.7). The WT, *Cyp27b1*-KO, and M1/M21-DIKO 0 IU all have 1,25(OH)_2_D_3_ levels at or below the LLOQ; however, the M1/M21-DIKO 20 IU diet shows an increase in 1,25(OH)_2_D_3_ levels (18.4 ± 5.4 pg/g; Figure 4H). While these biological replicates are variable, they were significantly increased, and the MSIs for the 1,25(OH)_2_D_3_ show punctate regions of higher ion intensity. The spleen and thymus both show lower levels of 25(OH)D_3_ that are at or below the LLOQ, and both tissues have an elevation of 25(OH)D_3_ in the M1/M21-DIKO mice on the 20 IU diet. Unlike the modest elevations in the 25(OH)D_3_, the spleen had significant increases in 1,25(OH)_2_D_3_ in both M1/M21-DIKO dietary samples (WT, <LLOD; C27-KO, 5.4 ± 0.8 pg/g; DIKO 0 IU, 16.3 ± 2.3 pg/g; DIKO 20 IU, 57.8 ± 4.7 pg/g; Figure 4H), and the MSIs depict a broad elevation of ion intensity of metabolite across the tissue. In Figure 4I, we examined the spatial features of MSI. The splenic data (Figure 4E) were overlaid with an H&E staining of a section taken immediately following the MSI tissue section. We found that, while 25(OH)D_3_ was rather broadly increased, it appears that the 1,25(OH)_2_D_3_ intensity increased in the white pulp segments of the thymus, which are rich in T cells and macrophages that encompass the periarteriolar sheaths, follicles, and marginal zones (25). Finally, 1,25(OH)_2_D_3_ in the thymus was also increased modestly with DIKO 20 IU diet (WT, 5.5 ± 0.9 pg/g; DIKO 20 IU, 16.9 ± 1.7 pg/g; Figure 4, F and H). These data indicate that these nonrenal tissues, and possibly others not examined, are both (a) transporting 25(OH)_2_D_3_ into these tissues and (b) may be leading to the elevation of 1,25(OH)_2_D_3_ detected in the plasma by LC-MS/MS analysis (Figure 4B).

**Figure 5:**
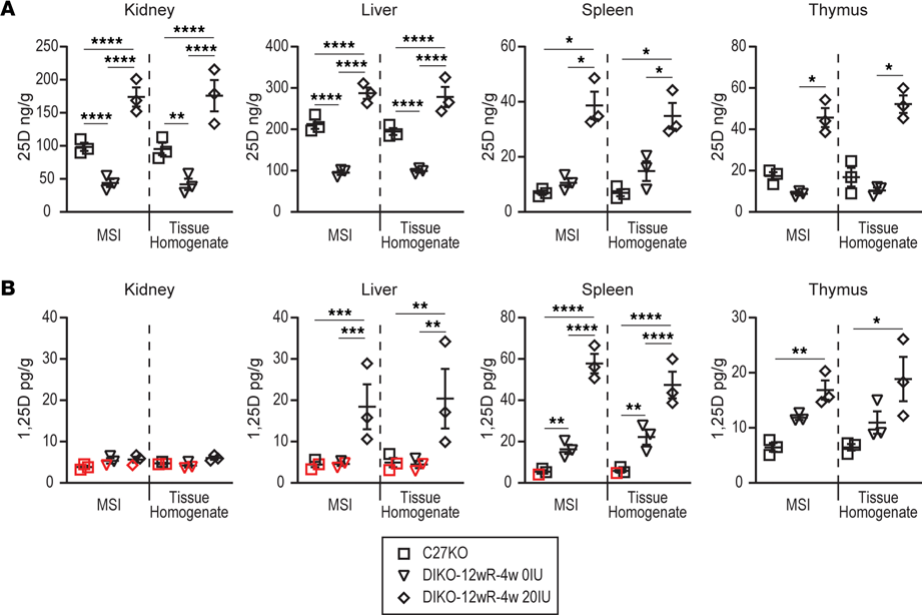
Confirmatory vitamin D metabolite levels in tissue homogenate. (**A** and **B**) LC-MS/MS was performed on the remaining tissue not used for sectioning for 25(OH)D_3_ (**A**, 25D, ng/g) and 1,25(OH)_2_D_3_ (**B**, 1,25D, pg/g) in the *Cyp27b1*-KO (C27-KO), M1/M21-DIKO 12wR-4w 0 IU, and M1/M21-DIKO 12wR-4w 20 IU. Absolute quantitation of biological triplicates is shown for 25(OH)D_3_ (**A**, ng/g) and 1,25(OH)_2_D_3_ (**B**, pg/g) for each tissue compared with the MSI values from Figure 4 (all values in Supplemental Figure 1). Data points that fall below the lower limits of quantitation (<LLOQ) but above the LLOD are shown in red. *n* = 3 for all tissues. One-way ANOVA (genotypes within tissues) with multiple comparison Tukey post hoc test. *P < 0.05; **P < 0.01; ***P < 0.001; ****P < 0.0001. There were no differences between groups in MSI versus tissue homogenate in all panels (by 2-way ANOVA and 1-tailed *t* test).

To confirm the validity of the MSI relative quantitation data for each of these tissues, we conducted an important homogenate analysis of the remaining tissue block by LC-MS/MS absolute quantitation. We processed the *Cyp27b1*-KO (chow) and M1/M21-DIKO (0 IU and 20 IU rescue diet) samples for this confirmation. 25(OH)D_3_ levels from the tissue homogenate mirrored those from the MSI data analysis (Figure 5A; example: C27-KO – MSI, 98.5 ± 6.1 ng/g versus tissue homogenate, 95.3 ± 9.4 ng/g, remaining data found in Supplemental Figure 1). Similarly, data for 1,25(OH)_2_D_3_ (Figure 5B) corroborate their MSI counterparts well, thus confirming the validity of the MSI data. In further validation of the method, a Bland-Altman analysis was performed (examples shown in Supplemental Figure 4) (26). Despite the limited *n* values (n=3), this analysis shows an average bias of 1.013 ± 0.121, and the 95% limits of agreement ranged from 0.85 ± 0.06 to 1.21 ± 0.12 across each grouping of samples between the methods, indicating that these methods are highly comparable.

### Gene expression consequences of elevated tissue 1,25(OH)_2_D_3_.

**Figure 6:**
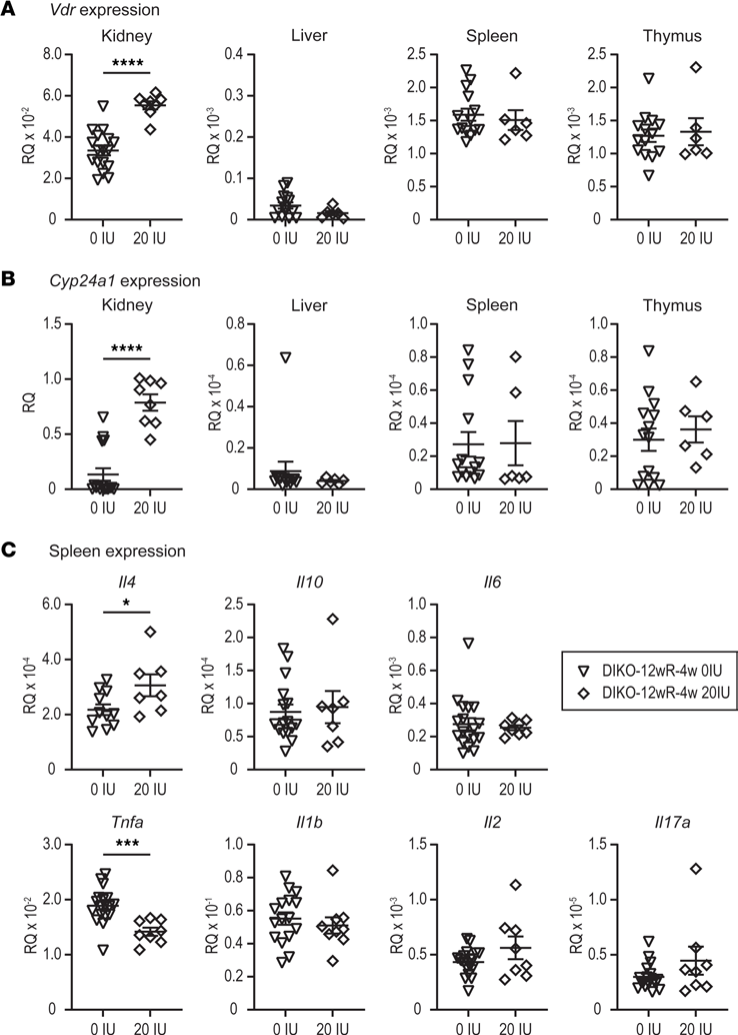
Gene expression changes in response to tissue elevations of 1,25(OH)_2_D_3_. (**A**–**B**) Gene expression was performed in the kidney, liver, spleen, and thymus for mice on the M1/M21-DIKO 12wR-4w 0 IU (*n* ≥ 14), and M1/M21-DIKO 12wR-4w 20 IU) *n* ≥ 6) for *Vdr* (**A**) and *Cyp24a1* (**B**). (**C**) Cytokine gene expression was measured in the spleen for *Il4*, *Il10*, *Il6*, *Tnfa*, *Il1b*, *Il2*, and *Il17a* in the same diet conditions as **A** and **B**. Unpaired *t* tests were performed. ****P < 0.0001; ***P < 0.001; *P < 0.05, 20 IU versus 0 IU.

With elevated 1,25(OH)_2_D_3_ expression in tissues, we hypothesized that these tissues may have elevated levels of vitamin D target genes. We first confirmed the elevation of megalin and cubulin expression in the kidney versus nonrenal tissues (Supplemental Figure 5). In Supplemental Figure 5, there was 100-fold more megalin (*Lrp2*) and cubulin (*Cubn*) found in the kidney, so 25(OH)D_3_ availability may be the highest. Furthermore, for these target genes to be responsive to 1,25(OH)_2_D_3_, the tissue should have expression of the vitamin D receptor (VDR). Therefore, we also evaluated the levels of *Vdr* expression in each of the tissues examined by MSI and in the M1/M21-DIKO mice that were fed either the 12-week rescue diet followed by 4 weeks of 0 IU or by 4 weeks of 20 IU. As can be seen in Figure 6A, the kidney had the highest expression of *Vdr*, and this expression was increased with the feeding of the 20 IU diet. The liver showed 100-fold lower *Vdr* expression than the kidney, and the spleen and thymus *Vdr* expression were both 10-fold lower and unchanged in either diet condition. We next wanted to assess the downstream consequences of higher 1,25(OH)_2_D_3_ production in the tissues by looking at the target gene of *Cyp24a1*, one of the most sensitive vitamin D target genes. As can be seen in Figure 6B, the kidney retained the highest level of *Cyp24a1* expression that was increased by the 20 IU diet. The remaining tissues were 1,000-fold lower in expression and were unchanged by diet. The expression of *Cyp24a1* was at the threshold of detection by quantitative PCR (qPCR) analysis in all tissues outside of the kidney in the absence of exogenous 1,25(OH)_2_D_3_ (9). It appeared that either the elevated plasma levels of 1,25(OH)_2_D_3_ in the 20 IU diet (Figure 4B and Supplemental Figure 1) may have been causing an elevation of *Cyp24a1* and *Vdr* in the kidney or that the retention of 25(OH)D_3_ in the kidney had reached levels high enough to activate the VDR directly to increase both *Cyp24a1* and *Vdr* (24, 27–29). The expression of *Cyp27b1* was unchanged in any diet condition (data not shown).

If the hypothesis of localized 1,25(OH)_2_D_3_ production affecting the inflammatory program of the immune cells is true, then we may see differential regulation of cytokines involved in pro- and antiinflammatory responses (2, 30). In Figure 6C, we examined the gene expression of several antiinflammatory cytokines (*Il4*, *Il10*) and proinflammatory cytokines (*Tnfa*, *Il17b*, *Il6*, and *Il1b*) in the spleen, which was the tissue with the highest 1,25(OH)_2_D_3_ concentration demonstrated by both MSI and confirmatory tissue homogenate. 1,25(OH)_2_D_3_ has been shown to suppress the proinflammatory cytokines like *Tnfa* and *Il17b* and increase antiinflammatory cytokines like *Il4* and *Il10* in macrophages and T cells during polarization, inflammatory disease, and cell differentiation (31–34). We found that *Il4* was significantly increased in the spleen, whereas *Tnfa* was suppressed. The remaining cytokines were not statistically changed. These data demonstrate a potential outcome of the production of 1,25(OH)_2_D_3_ in the spleen.

## Discussion

The potentially novel finding of this study is the detection and measurement of nonrenal vitamin D metabolite production in the absence of circulating 1,25(OH)_2_D_3_. This was only made possible by using our M1/M21-DIKO animal and its unique phenotype that mirrors the *Cyp27b1*-KO model while retaining a mature CYP27B1 protein (all tissues) and nonrenal inflammatory induction of *Cyp27b1* (11). A human study of anephric patients was recently published that corroborate our findings on the potential for nonrenal 1,25(OH)_2_D_3_ production in the absence of kidney 1,25(OH)_2_D_3_ production (17). Our unique animal model gives us the flexibility to manipulate dietary conditions, model different diseases, and isolate tissues for direct measurements. Here, we isolated the ability of vitamin D_3_ supplementation to elevate tissue distribution of both 25(OH)D_3_ and 1,25(OH)_2_D_3_ in the animal and to assess the downstream consequences after our dietary rescue. Using our targeted-metabolite MSI platform, we were able to identify and confirm tissue distribution of 25(OH)D_3_ and 1,25(OH)_2_D_3_ in the WT mice and the control animal, the *Cyp27b1*-KO mouse. Interestingly, the relative quantitation revealed that the WT mouse contained approximately 3-fold higher levels of 1,25(OH)_2_D_3_ in the tissue (66.4 ± 3.4 pg/g; Figure 4H and Supplemental Figure 1) compared with 1,25(OH)_2_D_3_ measured in the plasma (23.7 ± 1.9 pg/mL; Figure 4B and Supplemental Figure 1). The 25(OH)D_3_ levels, on the other hand, were also elevated in the kidney tissue (34.1 ± 3.1 ng/g; Figure 4A and Supplemental Figure 1) versus the plasma measurements (15.4 ± 1.1 ng/mL; Figure 4G and Supplemental Figure 1). This provides an interesting observation and perhaps an avenue to explore the export of 1,25(OH)_2_D_3_ from the tissue into the plasma, or it may be a consequence of the degradation of 1,25(OH)_2_D_3_ (or 25[OH]D_3_) to 1,24,25(OH)_2_D_3_ (or 24,25[OH]_2_D_3_) prior to exit from the tissue. 1,24,25(OH)_2_D_3_ and 24,25(OH)_2_D_3_ were not measured in this study but are a focus for future experiments.

Outside of the kidney, we expected the liver to be a repository of 25(OH)D_3_, and it was, with levels that were elevated from the plasma (35, 36). The thymus and spleen had very little 25(OH)D_3_ and levels of 1,25(OH)_2_D_3_ (Figure 4) that were at or below the LLOQ or LLOD on a diet with normal vitamin D levels; this was our expectation. The *Cyp27b1*-KO mice were an excellent control and recapitulated the expectations from the plasma analysis of vitamin D metabolites. The *Cyp27b1*-KO mice are known to have elevated levels of 25(OH)D_3_, and this was apparent in both in the liver and kidney tissues (10). This elevation is due to 2 phenomena: (a) inactivation of the CYP27B1 enzyme — thus, no conversion — and, more importantly, (b) secondary hyperparathyroidism that suppresses the activity and expression of the *Cyp24a1* gene and CYP24A1 enzyme (9, 11). The levels of 1,25(OH)_2_D_3_ are 1,000-fold less than 25(OH)D_3_ in the plasma (picograms versus nanograms); thus, the driving force behind the substrate excess is likely more due to this suppression of *Cyp24a1* and the inability to degrade 25(OH)D_3_ to 24,25(OH)_2_D_3_. Our previous studies show that, when PTH levels are high, there is very little conversion to 24,25(OH)_2_D_3_ (9–11). The MSI captures these features faithfully to previous observations as well as highlights the fact that, while plasma levels of 1,25(OH)_2_D_3_ are easiest to measure clinically, the tissue levels appear to be a significant variable and may vary widely in patient populations. Additionally, the ratios of 25(OH)D_3_ to not only 24,25(OH)_2_D_3_ but also to the production of the 25(OH)D_3_-26,23-lactone, a metabolite that is in less flux than 24,25(OH)_2_D_3_, are valuable in determining output of vitamin D metabolism in clinical patients (37–39). These additional metabolites will be examined in future studies.

Based on our previous skeletal rescue in the M1/M21-DIKO mice (11), we chose to first establish a rescued animal in both the skeleton and PTH levels in the plasma prior to changing the vitamin D levels in the diet. We had previously optimized this diet for skeletal rescue over a minimum of 12 weeks (11). We then changed the diet for an additional 4 weeks with varying levels of vitamin D_3_ supplementation, selecting the 0 IU diet and 20 IU diet as the extremes. We chose the 20 IU diet based on prior studies that demonstrated increased 25(OH)D_3_ availability in tissues and high elevation of serum 25(OH)D_3_ after 12 weeks of feeding (24) to ensure that transport differences in the tissues, based on megalin/cubulin expression, were overcome to deliver 25(OH)D_3_ into the nonrenal tissues. At the 4-week time point, the mice on the 0 IU diet were not vitamin D “deficient,” as evidenced by the plasma levels of 25(OH)D_3_ measured (Supplemental Figure 1). However, with extended feeding of this diet, we have achieved plasma 25(OH)D_3_ levels below the LLOD by LC-MS/MS absolute quantitation (data not shown). The 0 IU diet did achieve the stated purpose for our study at 4 weeks, as the MSI shows that tissue levels were greatly depleted of 25(OH)D_3_ in the kidney (Figure 3 and Figure 4). Elevation of vitamin D_3_ in the diet to 20 IU/g increased the 25(OH)D_3_ in the plasma, liver, and kidney. This elevation, though, did not lead to any more 1,25(OH)_2_D_3_ being made in the kidney of the M1/M21-DIKO mouse. Importantly, we found 25(OH)D_3_ by MSI in the spleen and modestly in the thymus, providing the substrate for potential conversion to 1,25(OH)_2_D_3_. In turn, we found that the 1,25(OH)_2_D_3_ in the spleen was elevated to levels that rivaled the WT kidney tissue (spleen 20 IU, 57.8 ± 4.7 pg/g; kidney WT, 66.4 ± 3.4 pg/g; Figure 4). It is possible, then, that the spleen is contributing to the elevated plasma levels of 1,25(OH)_2_D_3_ that are found in the M1/M21-DIKO mice on the 20 IU diet; however, there are many other tissues that may also be contributing in a similar manner that were not analyzed by MSI in this study, such as the parathyroid, skin, or intestinal tissues.

We also found the elevation in liver 1,25(OH)_2_D_3_ an intriguing result (Figure 4). The MSI shows punctate regions of increased ion intensity. The resolution of MSI does not yet allow cellular resolution, but it is possible that there are small cell populations like stellate cells or infiltrated macrophages resident in the liver that are causing these punctate ion intensity signals (40, 41). As mentioned, the current resolution of MSI is near 35 μm in size, thus ablating ~20–25 cells at a time, so this method is incapable of individual cell resolution. Similarly, the apparent low expression of *Cyp27b1* in nonrenal tissues could represent adequate expression in a select subpopulation of cells surrounded by a high-abundance, homogenous mix of nonexpressing cells. Other projects in the lab are focused on the discovery of these subpopulations. Finally, we are not proposing the 20 IU diet as a human clinical dietary intervention; it was chosen to portray a “high” or “maximal” effect in these studies to validate the MSI technique and dietary differences. Now that we have established this vital linkage between supplementation and nonrenal production of calcitriol, we will examine different vitamin D_3_ supplementation levels in diets and their outcome not only in MSI but also for inflammatory disease amelioration in M1/M21-DIKO, WT, and additional genotypes.

The power of MSI is in visualizing the spatial distribution of these vitamin D metabolites within the tissue themselves, in addition to the metabolite relative quantitation. We saw in Figure 2A, and from the schematic in Figure 2E and extrapolating that to Figure 2A, that the 25(OH)D_3_ appeared to be focused in the kidney cortex and outer medulla, whereas 1,25(OH)_2_D_3_ appeared across the cortex, outer medulla, and inner medulla, with the highest intensity apparent at the inner medulla. We find it interesting that 1,25(OH)_2_D_3_ had a higher intensity in what appears to be a different location than peak 25(OH)D_3_ intensity. Our previous genomic data indicate that there was an obvious tissue-specific isolation of the *Cyp27b1* enhancers to the proximal convoluted tubule cells (8). While these data are intriguing, we do not understand these distributions and why it appears the locations differ between the substrate and product. It is possible that some of this can be explained by the inevitable diffusion of metabolites during the OTCD processing and, thus, a technical aberration (23). It also highlights the need for continued development and experimentation in this powerful platform. Despite this, the MSI technique is clearly a methodology that can be used to study the transport of these vitamin D metabolites from tissues. For the spleen in Figure 4I, we found that the 25(OH)D_3_ was widely distributed, but the 1,25(OH)_2_D_3_ had higher relative ion intensity in what appeared to be the germinal centers and perhaps the marginal zones within the white pulp of the spleen (42). These areas contain higher concentrations of B cells under development and macrophages. Macrophages are 1 cell type that has been known to generate 1,25(OH)_2_D_3_ in an inflammatory disease state (12, 43, 44). We are now focused on refining this resolution and limiting diffusion to focus on specific structures, cell populations, and regions of interest. One such application will be in defining the contributions of vitamin D metabolites in immune cells or others at the point of disease contact in areas such as colonic or atherosclerotic lesions. While 25(OH)D_3_ and 1,25(OH)_2_D_3_ are important to identify, we are also focused on expanding this technique to capture the catabolic derivatives of these metabolites in 24,25(OH)D_3_; 1,24,25(OH)D_3_; and others. We and others have demonstrated relationships between metabolites such as 25(OH)D_3_ and 24,25(OH)D_3_ that can be predictive on PTH levels and skeletal health (37, 45, 46). As we expand our studies to disease models, we will explore the ratios of 25(OH)D_3_/24,25(OH)_2_D_3_ and 25(OH)D_3_/25(OH)D_3_-26,23-lactone in tissues compared with the plasma.

In the final set of experiments, we looked at the effect of 1,25(OH)_2_D_3_ production in the tissues on gene expression. For 1,25(OH)_2_D_3_ to have a consequence, the VDR should be expressed in these tissues. We confirmed from previous studies (10, 11) that the concentrations of *Vdr* expressed in the tissues we isolated in that kidney was the highest, followed by thymus and spleen, with the liver having very low levels of *Vdr* detected by qPCR (Figure 6). We also confirmed that *Cyp24a1* expression was very low in these nonrenal tissues, often at the threshold of detection by qPCR analysis. *Cyp24a1* was not elevated in these tissues after high vitamin D_3_ supplementation facing physiologic levels of 1,25(OH)_2_D_3_. We have found that supraphysiologic injections of 1,25(OH)_2_D_3_ easily activate the *Cyp24a1* gene in any tissue that contains the VDR (10, 11). This is different than the activities in the kidney after 20 IU vitamin D_3_ supplementation. Here in the kidney, the physiologic elevations in 1,25(OH)_2_D_3_ led to activation of *Cyp24a1*. We believe that this indicates 2 different modes for *Cyp24a1* activity in the body: (a) renal regulation of *Cyp24a1* responds to and maintains the circulating blood 1,25(OH)_2_D_3_ levels, and (b) nonrenal *Cyp24a1* exists for emergency detoxifying circumstances. When faced with physiologic levels of 1,25(OH)_2_D_3_, those nonrenal tissues preserve 1,25(OH)_2_D_3_ for positive gene expression (no *Cyp24a1* expressed); when exposed to higher levels of 1,25(OH)_2_D_3_, the detoxification program is initiated in all tissues. If vitamin D is antiinflammatory as the literature would suggest, we should see consequences of these 1,25(OH)_2_D_3_ changes in immune cells and in tissues that harbor immune cells like the spleen (2, 31, 34, 47). We did, in fact, see a change in both *Tnfa* and *Il4* in the spleen. These cytokines were unchanged in the liver and thymus (data not shown). In prior studies with *Cyp27b1*-KO mice, it was found that the 20 IU vitamin D_3_ dietary supplementation fed for 12 weeks was able to induce gene expression in the intestine (24). While we used a shorter term for vitamin D_3_ supplementation (4 weeks), and we found very little 25(OH)D_3_ in tissues outside of the kidney and liver, it is still possible these elevated levels of 25(OH)D_3_ are causing gene regulation in the spleen. 25(OH)_2_D_3_-actived VDR is very difficult to control for experimentally in the animal; therefore, we will continue to examine these diets at varying vitamin D_3_ concentrations to help clarify 25(OH)D_3_-activated VDR expression. Given our gene expression results in the spleen, it appears this elevation of splenic 1,25(OH)_2_D_3_ may affect the immune program. We are currently focused on isolation of different immune populations for closer examination of their expression and differentiation under these dietary conditions. We are also examining these cell populations in inflammatory disease models for their contribution to amelioration of disease.

In conclusion, this work is the first example to our knowledge of leveraging the power of the OTCD-MALDI-MSI platform to capture and quantify the renal and nonrenal production of vitamin D metabolites. We can manipulate the plasma levels of 25(OH)D_3_ through vitamin D_3_ supplementation, not 1,25(OH)_2_D_3_ or 25(OH)D_3_ dosing, and this appears to have consequences to gene expression and production of 1,25(OH)_2_D_3_. Continued development of this platform will enable us to identify the connection between plasma vitamin D levels, or status, and disease benefits that we believe will help guide future clinical recommendations.

## Methods

### Sex as a biological variable.

For all studies, both male and female mice were included in the analyses. Sex was considered as a biological variable, and data were reported as mixed where no differences were found in the data.

### Materials.

1α,25(OH)_2_D_3_, acetone, hexane, zinc sulphate, gelatine, 2-mercaptobenzotiazole (MBT), and sodium borohydride we purchased from Sigma-Aldrich. 25(OH)D_3_ commercially available lyophilized 4-point calibration standards were purchased from (Chromsystem). Amplifex Dieen reagent was purchased from Sciex (Chemistry and Consumables R&D). Isotopically labeled d6-25(OH)D_3_ and d6-1α,25(OH)_2_D_3_ were purchased from Cambridge Isotope Laboratories. 25(OH)D_3_ serum calibrators were purchased from Chromsystem GmbH. LC-MS grade acetonitrile (ACN), methanol, and formic acid were obtained from Thermo Fisher Scientific. The water used was generated through a direct-Q Ultrapure water system from MilliporeSigma with a specific resistance of 18.2 MΩ cm. Traditional genotyping PCR was completed with GoTaq (Promega), and all qPCR was completed with the StepOnePlus using TaqMan for gene expression assays (Applied Biosystems). Primers were obtained from IDT.

### Animal studies.

All mice were derived from or created in the C57BL/6J genetic background as previously described (10, 11). *Cyp27b1*-KO, M1/M21-DIKO, and littermate WT mice aged 8–9 weeks (The Jackson Laboratory) were housed in high-density ventilated caging in the Biochemistry Animal Research Facility of the University of Wisconsin-Madison under 12-hour light/dark cycles at 72°F and 45% humidity. Mice used in this study were maintained on a standard rodent chow diet (5008, Lab Diet), rescue diet (1.5 IU vit D/g, 2% Ca, 1.25% P, 20% lactose, TD.96348, Inotiv/Envigo), 0 IU diet (0 IU vit D/g, 2% Ca, 1.25% P, 20% lactose, TD.200097, Inotiv/Envigo), or 20 IU diet (20 IU vit D/g, 2% Ca, 1.25% P, 20% lactose, TD.200096, Inotiv/Envigo). M1/M21-DIKO mice were fed the rescue diet for 12 weeks after wean, followed by a diet switch to either the 0 IU or 20 IU diet for an additional 4 weeks (16 weeks total diet, mouse aged 19 weeks). Unless otherwise indicated, all experiments were conducted with equal numbers of males and females (*n* ≥ 6). From these mice, a subset was chosen for MSI (*n* = 3) for each diet and genotype condition, including both male and female in the groups. MSI is a technically complex method that is not easily scaled to high *n* value experiments. We determined that *n* = 3 provided statistically sound data for this study. Data were reported as mixed, as no differences were found between the sexes.

### Tissue perfusion and collection.

Mice were anesthetized under ether and opened, and blood was collected by cardiac puncture and split into serum or EDTA-treated plasma. Post puncture 10 mL of 1× PBS was perfused to remove blood from the circulation and tissues. Intratissue systemic contribution of blood in all target tissues was assessed by monitoring the Heme B ion from myoglobin containing an iron ion in the +3 oxidation state as (Fe[III] + porphyrin [C_34_H_32_N_4_O_4_Fe_1_])+ at *m/z* 616.1772 ± 0.025 Da. No systemic contribution was observed for all tissues as signal/noise (S/N) of the Heme B ion was below 3. For MSI, tissues were dissected, weighed, and drop frozen in liquid N_2_ in 2.0 mL cryo vials. For gene expression, tissues were dissected and drop frozen in liquid N_2_ in microfuge tubes. All tissues were stored long term in –80°C.

### Blood chemistry.

Cardiac blood was collected at the time of sacrifice. Collected blood was split into serum or EDTA-treated plasma and incubated at room temperature for 30 minutes followed by centrifugation at 3,400*g* for 12 minutes (×2) to obtain serum or EDTA-plasma (10). Serum Ca and P levels were measured using QuantiChrom Calcium Assay Kit (DICA-500, BioAssay Systems) and QuantiChrom Phosphate Assay Kit (DIPI-500, BioAssay Systems) (10). Circulating intact FGF23 and PTH were measured in EDTA plasma via a mouse/rat FGF-23 (intact) ELISA Kit (60-6800, Immutopics) and a mouse PTH (1-84) ELISA kit (60-2305, Immutopics), respectively (10).

### BMD.

BMDs were measured and analyzed by dual x-ray absorptiometry (DEXA) with a PIXImus2 densitometer (GE-Lunar Corp.) as previously described (48).

### Gene expression.

Frozen tissues were homogenized in Trizol Reagent (Invitrogen), and RNA was isolated as per the manufacturer’s instructions. In total, 1 μg of isolated total RNA was DNase treated, reverse transcribed using the High-Capacity cDNA Kit (Applied Biosystems), and then diluted to 100 μL with RNase/DNase-free water. qPCR was performed using primers specific to a select set of differentially expressed genes by TaqMan analyses. TaqMan Gene Expression probes (Applied Biosystems) were used (Supplemental Table 1).

### Tissue sectioning and mounting.

Tissue sectioning was performed on frozen target tissues from each mouse using a Leica cryostat (CM 1850 UV; Leica Biosystems) with gelatine (10% v/v) as a mounting medium. Adjacent cross sections (12 μm thickness) were taken from a top-down (horizontal) plane and thaw-mounted onto conductive indium tin oxide–coated (ITO-coated) slides (Bruker Daltonics GmbH & Co. KG). Further adjacent sections (10 μm thickness) were mounted onto a slide precoated with poly-l-lysine for histological staining. The remaining tissue was used for tissue homogenate analysis by LC-MS/MS. All sections were dried in a vacuum desiccator at room temperature for 30 minutes and stored at −80°C for analysis.

### Instrumentation.

MSI experiments were performed on an Orbitrap LTX-XL Mass Spectrometer (Thermo Fisher Scientific) couple to a reduced pressure ESI/MALDI interface (Spectroglyph LLC). Confirmatory LC-MS/MS analysis was performed using a triple-quadrupole linear Ion trap Mass Spectrometer SCIEX 5500+ Qtrap (Sciex) coupled with an Ultra high pressure Liquid Chromatography system Nexera series ultra high performance liquid chromatography (UHPLC) (Shimadzu).

### OTCD.

At –80°C, tissue sections were dried in a vacuum desiccator (20 minutes). Amplifex reagent (5 mL, 0.1 mg/mL in 50:50 v/v ACN/water) was applied to the tissue sections by protocol adapted from ref. 23 using an artistic airbrush (AirFlow) instead of an automated Image Prep due to restricted quantities of Ampiflex reagent. Briefly, a reagent solution of Amplifex (2 mL, 0.25 mg/mL in 50:50 v/v ACN/water) with the incorporation of 10 μL of ITSD (d6-25[OH]D_3_ at 1 ng/mL was sprayed positioned 20 cm away from the target with a N_2_ pressure of 1.2 bar using 2 mL/slide, with each manual pass lasting approximately 1 second and repeated with 5–10 seconds between passes until uniform coating was achieved (ƍ of 0.015 mg/cm^2^). Then, the slide was placed in a closed reaction container, containing a 1:1 v/v solution of ACN/water and a moist Kimwipe under the lid and placed in an oven at 37°C for 1 hour for derivatization completion. Sections were then dried under vacuum desiccator (25 minutes) and were ready for MALDI matrix application.

### Matrix application.

Matrix application of 2-mercaptobenzothiazole (10 mL, 6 mg/mL in 90:10 v/v ACN/water) was applied in 8 passes using a 3D printer, as previously described (23, 49). A flow rate of 0.1 mL/min with a gas pressure of 2 bar, a bed temperature of 30°C, a *Z*-height of 30 mm, and a velocity of 1,100 mm/min were achieved, averaging a run time of 30 minutes per slide. A uniform coating of matrix was achieved with 0.12 mg/cm^2^.

### MSI acquisition.

MSI experiments were performed on an Orbitrap LTQ-XL Mass Spectrometer (Thermo Fisher Scientific) coupled with a reduced pressure Electrospray (ESI)/MALDI interface (Spectroglyph). Further details on the interface can be found in Belov et al. (50). The 349 nm MALDI laser (Spectra Physics) was operated at a repetition rate of 1,000 Hz and pulse energy of ~2.3 μJ. The mass spectrometer was operated in positive ion mode using a maximum ion injection time of 250 ms in FT mode, automatic gain control (ACG) turned off and a mass range of 500–800 Da. The spectral resolution was set to 60.000 full width at half maximum (FWHM) at *m/z* 400. The experiments were performed at 100 × 100 μm spatial resolution for liver and thymus tissue sections and 75 × 75 μm for kidney and spleen tissue sections. Optical images were taken using a flatbed scanner (Cannon LiDE-20). The instrument was calibrated using ESI Cal mix solution as per manufacture specifications. Collision-induced dissociation (CID) experiments on both vitamin D metabolites (1α,25[OH]_2_D_3_ and 25[OH]D_3_) Ampiflex derivatives was performed for structural confirmatory analysis at corresponding precursor masses *m/z* 732.50 and *m/z* 748.50 with an isolation window of 2 Da summing 10 scans/spectra using 16 and 17 V as collision energies (CE), respectively, covering a scan range of 280–750 Da.

### Spectra processing.

Thermo Fisher raw data were aligned to the corresponding position files in Image Insight software (Spectroglyph) and exported form there as imzML files using the supplier recommended default parameters. The imzML files were uploaded onto the open-source MSiReader version 1.02 9 using MATLAB version 2022b and Fleximaging version 5.1 (Bruker Daltonik). All data were normalized using the d6-25(OH)D_3_ mass at (molecular ion [M^+^]) 738.5435 ± 0.025 Da (peak normalization). MS images were generated for both vitamin D metabolites detected as Ampiflex derivatives at *m/z* 732.5058 ± 0.025 for 25(OH)D_3_ and *m/z* 748.5008 ± 0.025 for 1α,25(OH)_2_D_3_. For relative semiquantitation (tissue mimetic model), intensity values for each metabolite as Ampiflex derivatives were collected and ratios against the ISTD (d6-25[OH]D_3_) were calculated. Relative quantitation was accomplished by plotting ratio intensities against matrix-matched standard calibration using the mimetic model. MSiQuant (part of the MSiReader software package) was used as software for calculations.

### Confirmatory LC-MS/MS analysis (absolute quantitation).

Please see Supplementary Methods.

### Histological staining.

Please see Supplementary Methods.

### Statistics.

Data were analyzed using GraphPad Prism 10.2.2 software (GraphPad Software) and in consultation with the University of Wisconsin Statistics Department. All values are reported as the mean ± SEM, and differences between group means were evaluated using 1-way ANOVA or Student’s *t* test (1-tailed) as indicated in the figure legends. Bland-Altman was performed in GraphPad Prism. *P* < 0.05 was considered significant.

### Study approval.

All animal studies were reviewed and approved by the Research Animal Care and Use Committee of University of Wisconsin-Madison under protocol no. A005478.

### Data availability.

Values for all data points and graphs for this study are reported in the Supporting Data Values file and are included in the supplemental materials. All images used and replicates are available in the supplemental materials. Data from MSI studies were processed using MSiQuant software from the MSiReader Software Package and compiled in Excel and GraphPad Prism.

## Author contributions

Conceptualization was contributed by MBM, JWP, and DFC. Investigation was contributed by MBM, SML, SRC, and DFC. Writing of the original draft was contributed by MBM and DFC. Review and editing were contributed by MBM, SML, SRC, JWP, and DFC. Data curation was contributed by MBM.

## Supplementary Material

Supplemental data

Supporting data values

## Version 1 06/25/2024

In-Press Preview

## Version 2 08/08/2024

Electronic publication
