# Supplementary material for: Spatial detection and consequences of nonrenal calcitriol production as assessed by targeted mass spectrometry imaging
Source: JCI Insight. 2024 Jun 25;9(15):e181763. doi: 10.1172/jci.insight.181763 (PMC11383599; doi:10.1172/jci.insight.181763)
Supplement: Supplemental data [file jciinsight-9-181763-s231.pdf]

Figure S1

A Plasma LC-MS/MS

	ID	25D (ng/mL)	1,25D (pg/mL)
WT	101	13.4	22.5
	102	15.6	27.4
	103	17.1	21.1
C27KO	318	82.2	3.5
	319	72.5	4.1
	320	78.9	3.6
DIKO	218	61.4	5.7
	219	55.7	6.1
	220	70.3	5.2
12wR-4w 0 IU	273	46.5	6.5
	274	34.3	7.2
	279	27.1	4.2
12wR-4w 20 IU	296	78.5	15.6
	297	71.4	17.8
	302	88.8	20.1

B Lower Limits of Quantitation or Detection

	ID	25D	1,25D
Plasma	LLOQ	2 ng/mL	5 pg/mL
	LLOD	0.5 ng/mL	2 pg/mL
Tissue	LLOQ	5 ng/g	5 pg/g
	LLOD	2 ng/g	2 pg/g

C MSI relative quantitation

		Kidney		Liver		Spleen		Thymus	
	ID	25D (ng/g)	1,25D (pg/g)	25D (ng/g)	1,25D (pg/g)	25D (ng/g)	1,25D (pg/g)	25D (ng/g)	1,25D (pg/g)
WT	101	35.3	61.3	230.1	4.4	7.2	<LLOD	3.3	4
	102	28.2	72.7	192.2	5.6	6.1	<LLOD	4.2	5.3
	103	38.7	65.1	185.3	3.1	7.9	<LLOD	6.3	7.1
C27KO	318	95.6	3.8	235.4	4.5	6.8	5.2	13.5	6.5
	319	110.2	4.5	205.6	5.6	8.4	6.9	20.1	7.8
	320	89.7	3.2	197.1	3.3	5.7	4.1	18.4	5.1
12wR-4w 0 IU	273	43.5	5.2	101.8	4.8	10.5	15.9	8.8	11.5
	274	54.6	6.5	99.3	5.3	7.9	20.4	7.4	11.4
	279	33.3	4.3	84.7	3.7	13.4	12.6	10.1	12.7
12wR-4w 20 IU	296	168.7	5.9	287.9	15.8	34.8	56.1	44.1	15.6
	297	152.1	4.3	263.5	10.6	32.7	50.7	38.7	14.7
	302	201.2	6.7	311	28.9	48.6	66.6	54.3	20.3

D Tissue Homogenate LC-MS/MS

		Kidney		Liver		Spleen		Thymus	
	ID	25D (ng/g)	1,25D (pg/g)	25D (ng/g)	1,25D (pg/g)	25D (ng/g)	1,25D (pg/g)	25D (ng/g)	1,25D (pg/g)
C27KO	318	91.1	4.6	211.2	3.1	5.1	7.7	9	7.2
	319	113.2	5.1	188.3	7	9.3	5.2	24.6	5.3
	320	81.5	4.5	184.6	4.7	6.5	4.8	16.9	6.9
12wR-4w 0 IU	273	38.3	3.9	99.1	5.9	16	23.5	12.1	9.1
	274	57.9	5.1	105.7	4.6	8.2	27.8	11.5	8.8
	279	29.1	3.6	92.2	2.9	20.4	15.3	7.7	15
12wR-4w 20 IU	296	178.8	6.7	265.6	17.1	29.4	43.6	51.3	18.3
	297	133.4	5.4	244	9.9	31.1	38.7	45.4	12.2
	302	215.5	5.9	325.8	34.2	44.2	60	59.9	26.1

Figure S2

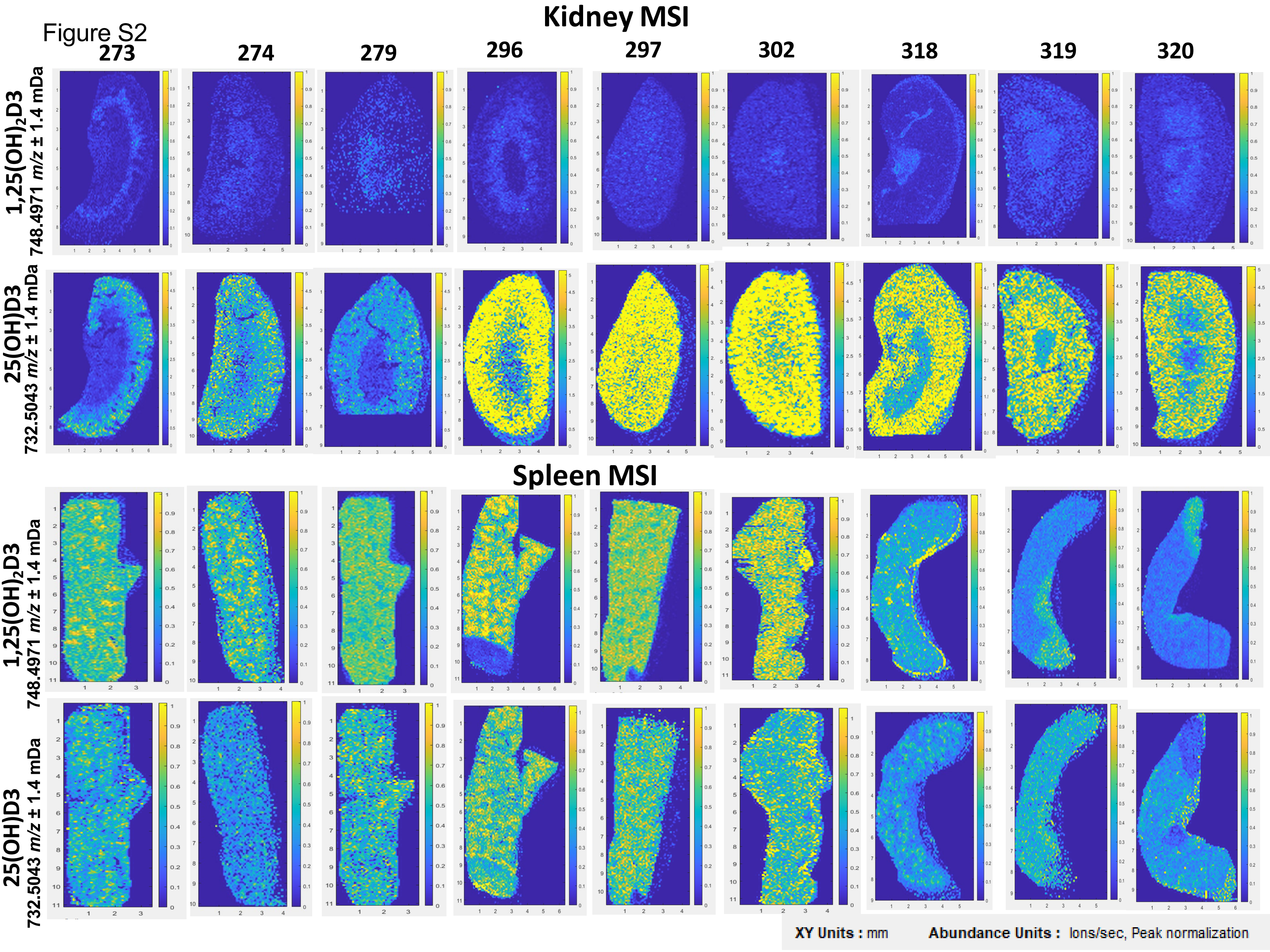
Kidney MSI
273
274
279
296
297
302
318
319
320
1,25(OH)2D3 748.4971 m/z ± 1.4 mDa
25(OH)D3 732.5043 m/z ± 1.4 mDa
Spleen MSI
1,25(OH)2D3 748.4971 m/z ± 1.4 mDa
25(OH)D3 732.5043 m/z ± 1.4 mDa
XY Units : mm
Abundance Units : Ions/sec, Peak normalization

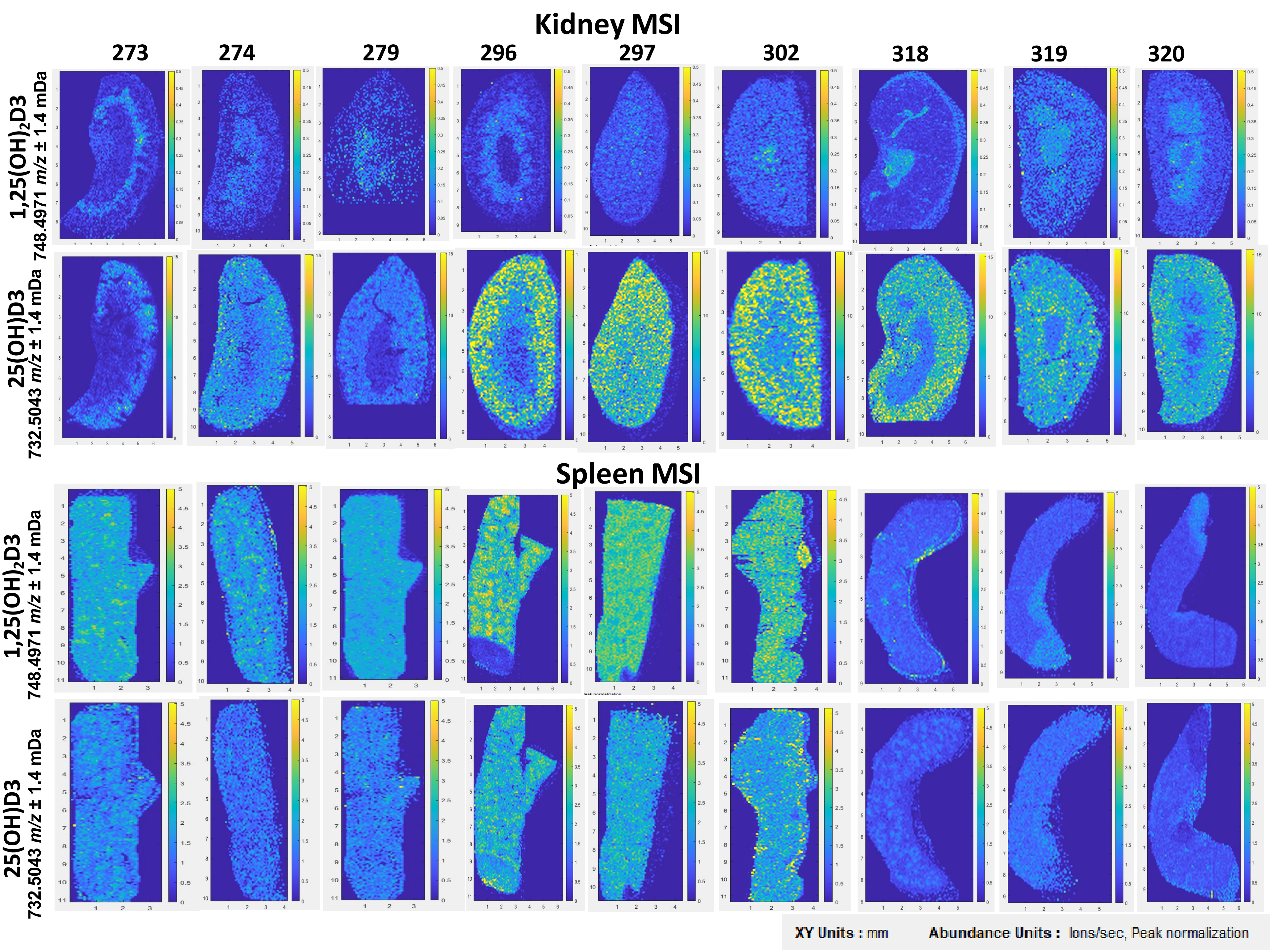
Kidney MSI
273
274
279
296
297
302
318
319
320
1,25(OH)$_2$D3 748.4971 m/z ± 1.4 mDa
25(OH)D3 732.5043 m/z ± 1.4 mDa
Spleen MSI
1,25(OH)$_2$D3 748.4971 m/z ± 1.4 mDa
25(OH)D3 732.5043 m/z ± 1.4 mDa
XY Units : mm
Abundance Units : Ions/sec, Peak normalization

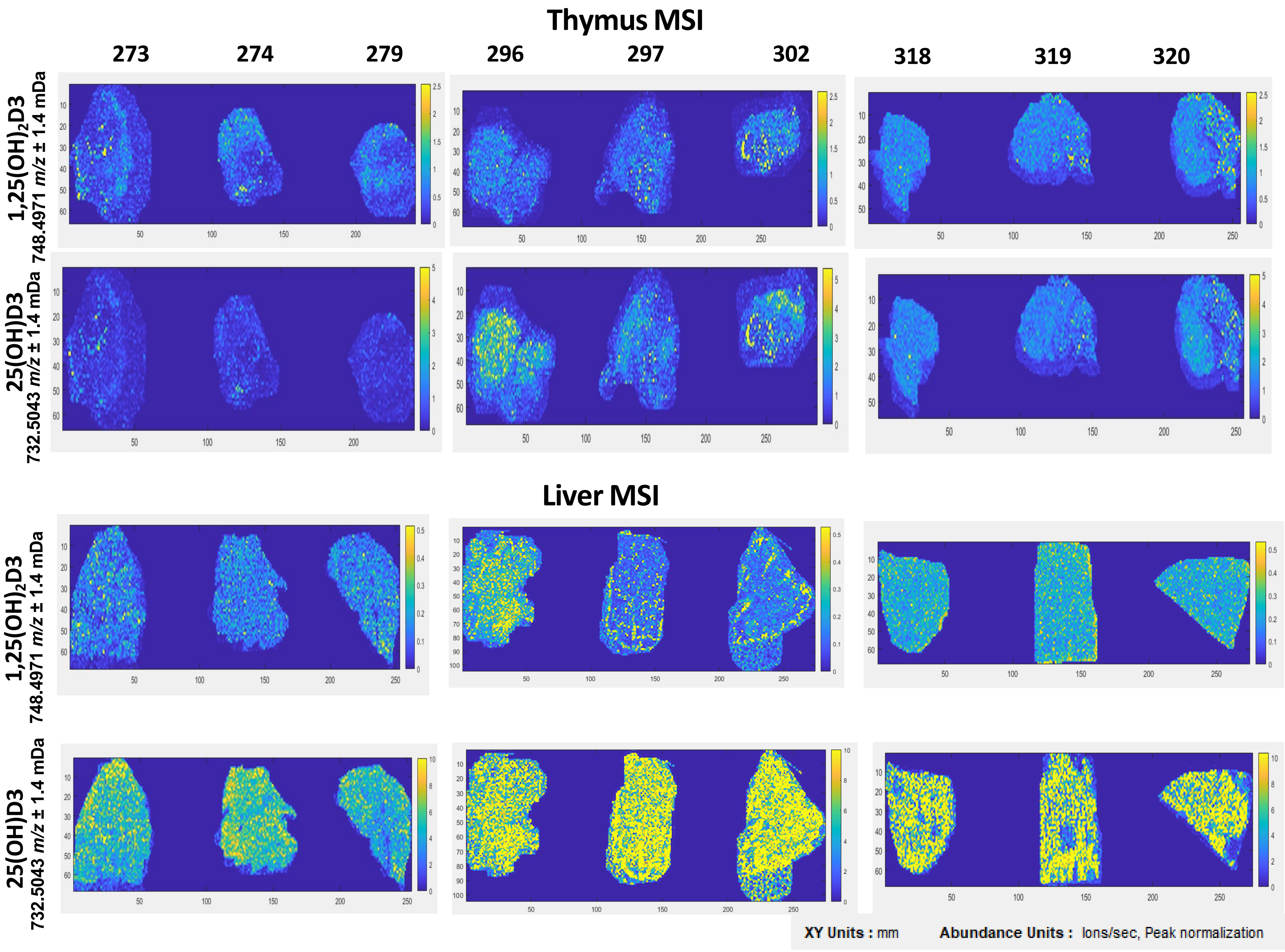
Thymus MSI
273
274
279
296
297
302
318
319
320
1,25(OH)$_2$D3
748.4971 *m/z* ± 1.4 mDa
25(OH)D3
732.5043 *m/z* ± 1.4 mDa
Liver MSI
1,25(OH)$_2$D3
748.4971 *m/z* ± 1.4 mDa
25(OH)D3
732.5043 *m/z* ± 1.4 mDa
XY Units : mm
Abundance Units : Ions/sec, Peak normalization

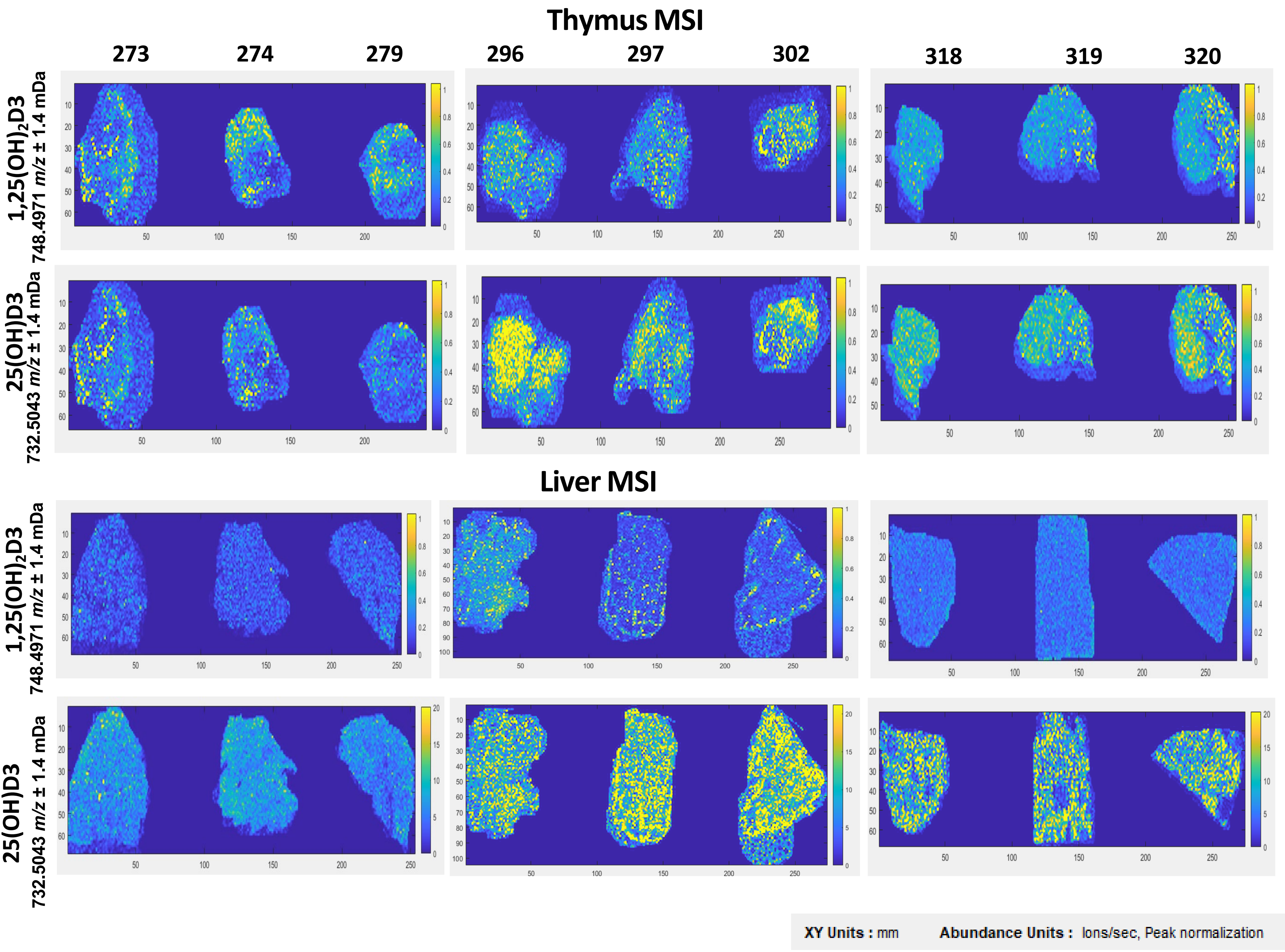

Thymus MSI
273
274
279
296
297
302
318
319
320
1,25(OH)2D3
748.4971 m/z ± 1.4 mDa
25(OH)D3
732.5043 m/z ± 1.4 mDa
Liver MSI
1,25(OH)2D3
748.4971 m/z ± 1.4 mDa
25(OH)D3
732.5043 m/z ± 1.4 mDa
XY Units : mm
Abundance Units : Ions/sec, Peak normalization

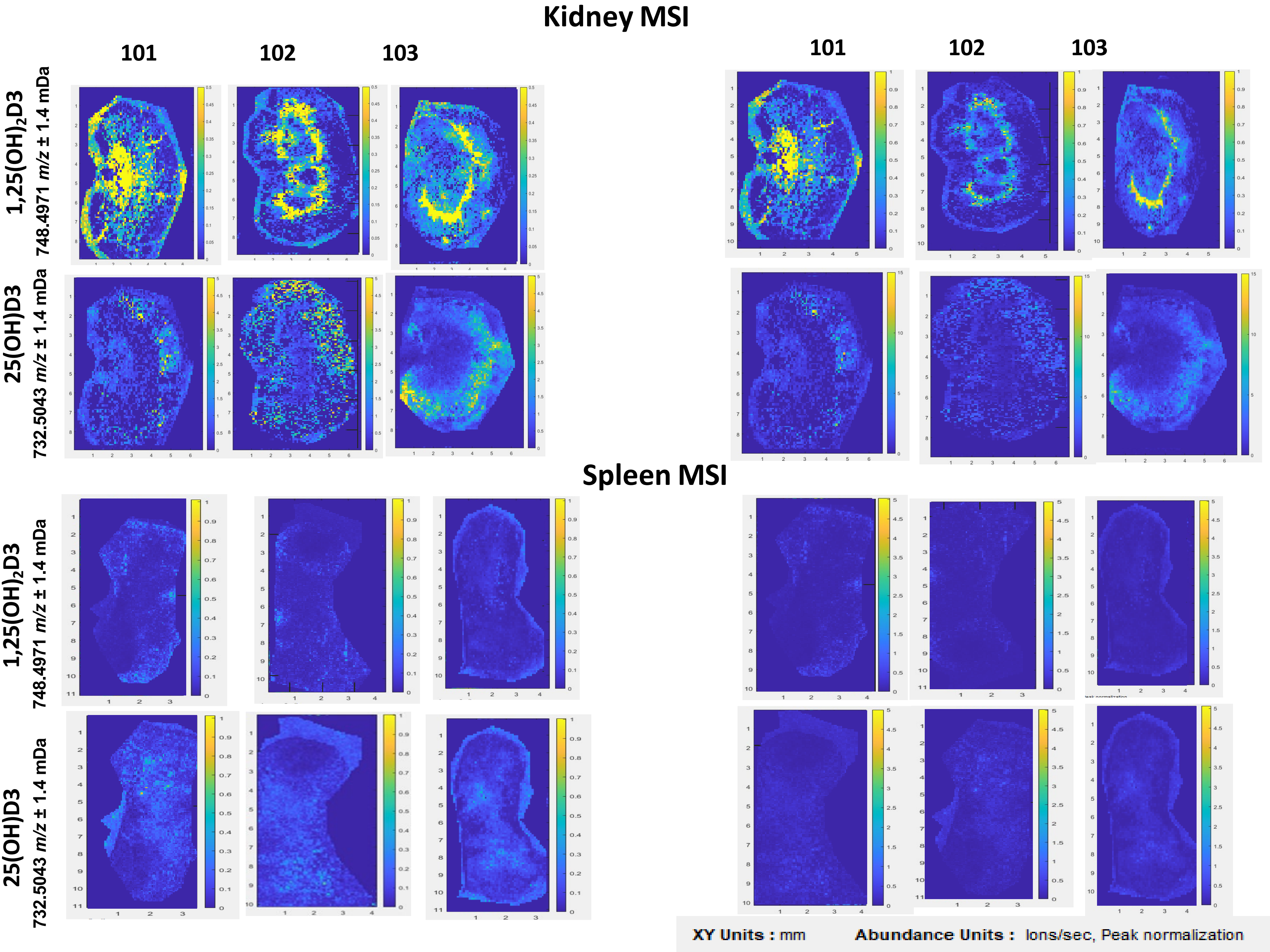
Kidney MSI
101
102
103
101
102
103
1,25(OH)2D3
748.4971 m/z ± 1.4 mDa
25(OH)D3
732.5043 m/z ± 1.4 mDa
Spleen MSI
1,25(OH)2D3
748.4971 m/z ± 1.4 mDa
25(OH)D3
732.5043 m/z ± 1.4 mDa
XY Units : mm
Abundance Units : Ions/sec, Peak normalization

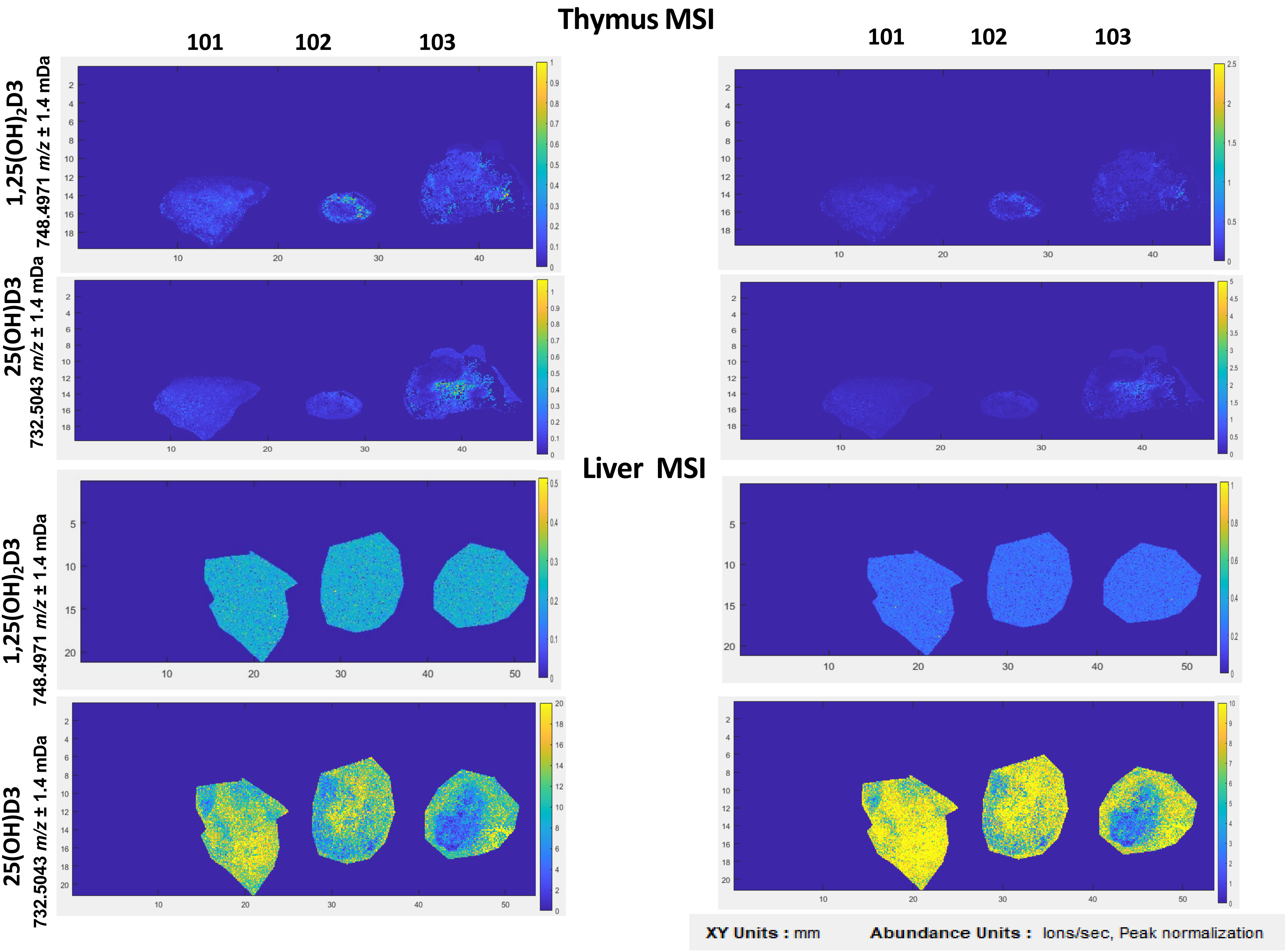

Thymus MSI
101
102
103
1,25(OH)2D3
748.4971 m/z ± 1.4 mDa
25(OH)D3
732.5043 m/z ± 1.4 mDa
Liver MSI
XY Units : mm
Abundance Units : Ions/sec, Peak normalization

Figure S3

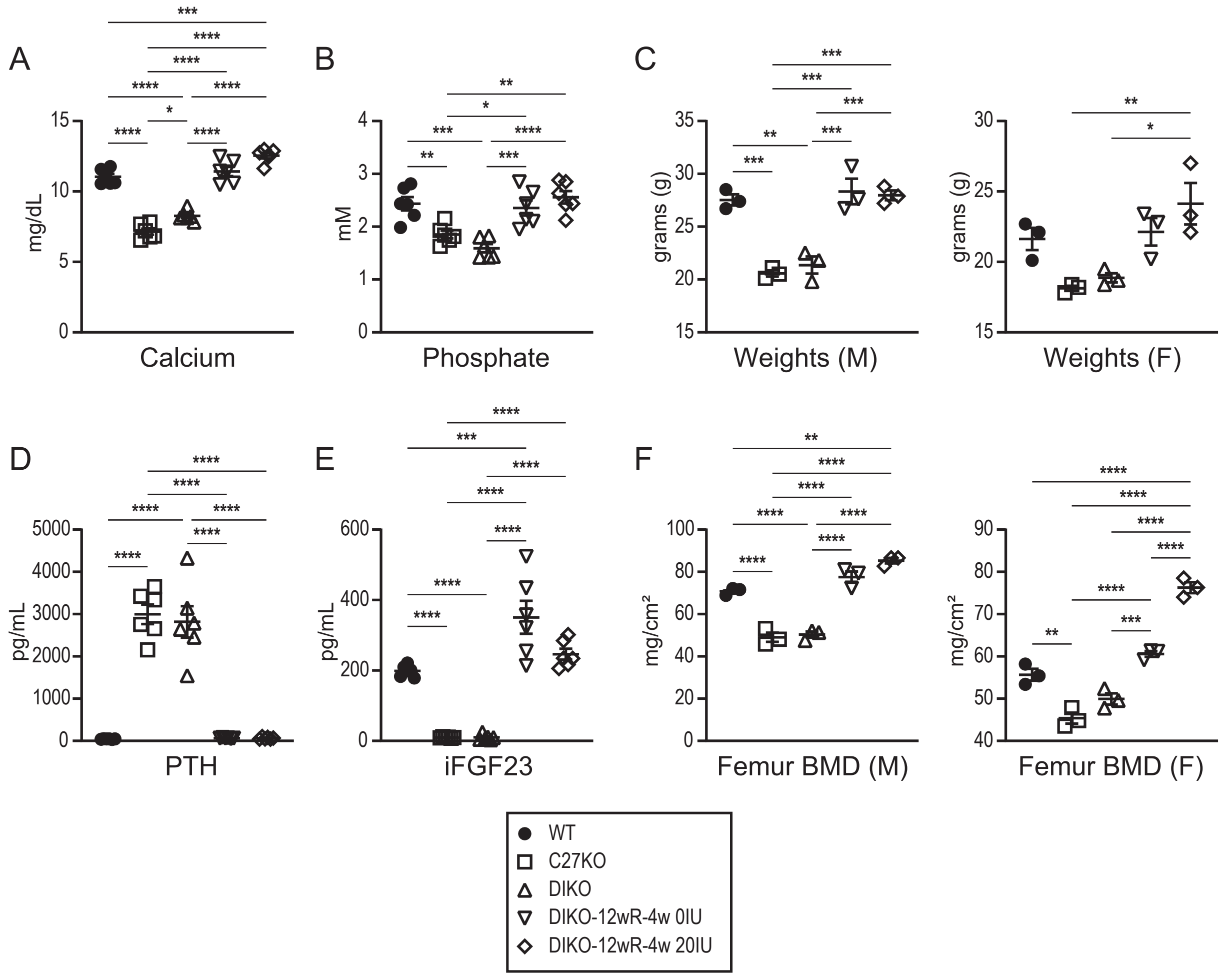
A
B
C
D
E
F
mg/dL
Calcium
mM
Phosphate
grams (g)
Weights (M)
Weights (F)
pg/mL
PTH
iFGF23
mg/cm²
Femur BMD (M)
Femur BMD (F)
WT
C27KO
DIKO
DIKO-12wR-4w 0IU
DIKO-12wR-4w 20IU

Figure S4

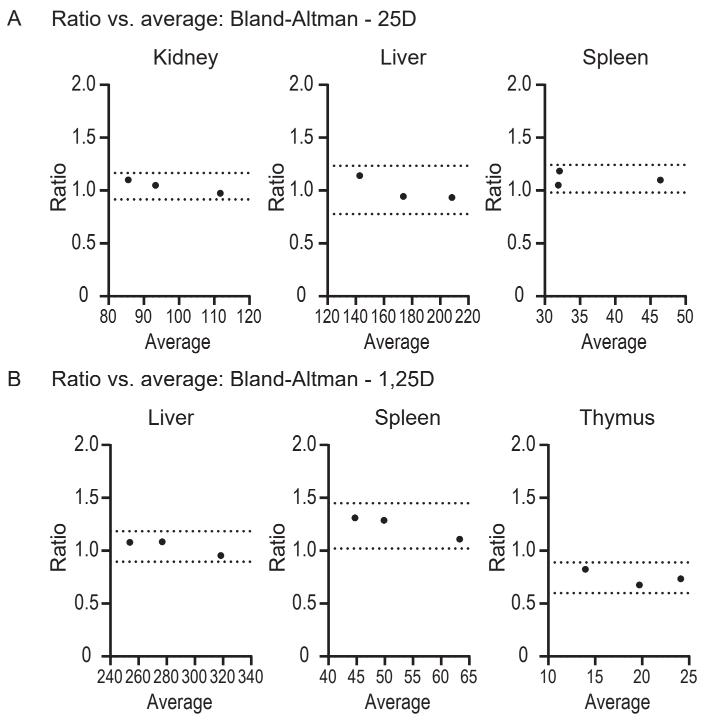
A
Ratio vs. average: Bland-Altman - 25D
Kidney
Liver
Spleen
Ratio
Average
B
Ratio vs. average: Bland-Altman - 1,25D
Liver
Spleen
Thymus
Ratio
Average

Figure S5

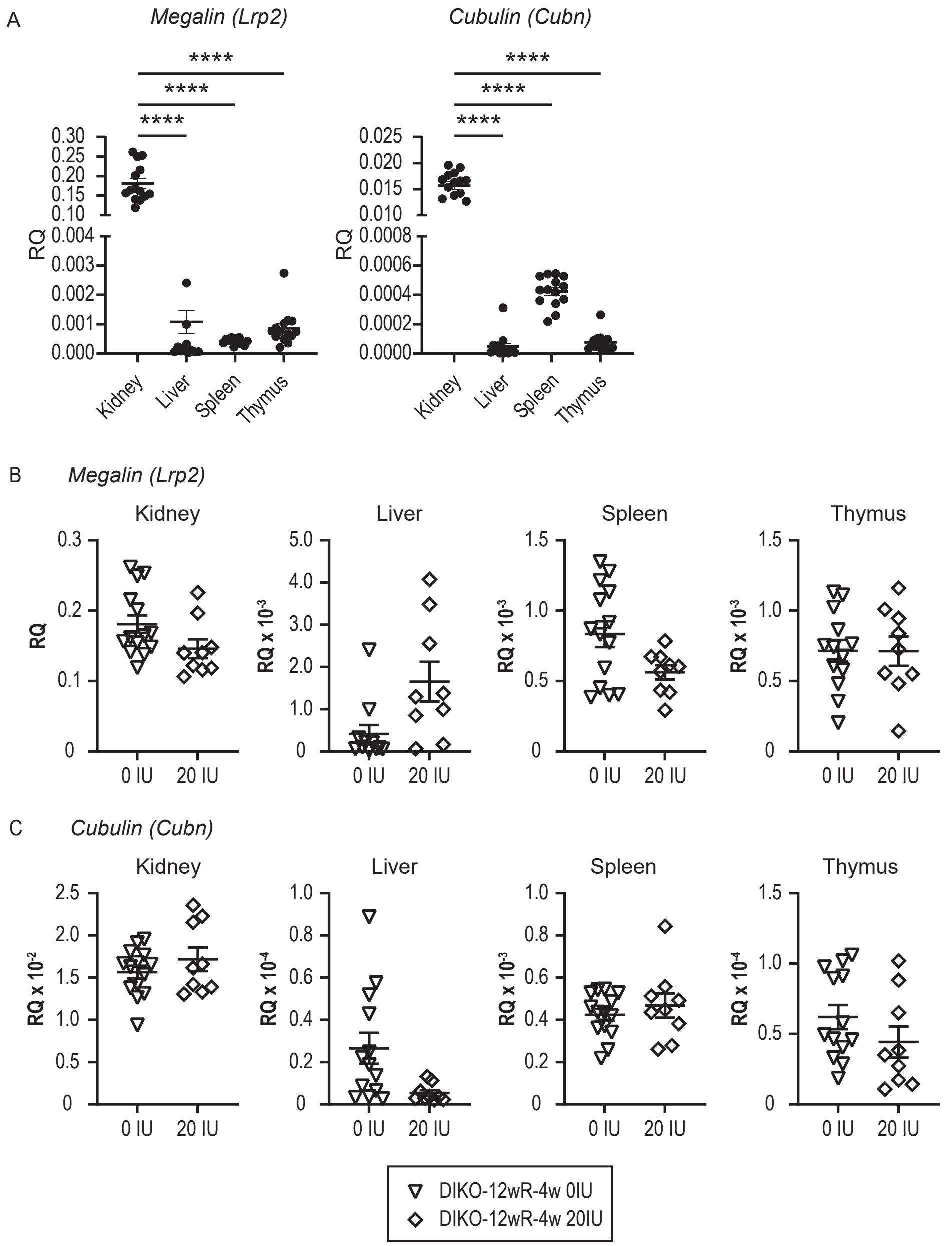
A
Megalin (Lrp2)
Cubulin (Cubn)
****
RQ
Kidney
Liver
Spleen
Thymus
B
Megalin (Lrp2)
C
Cubulin (Cubn)
0 IU
20 IU
RQ x 10-3
RQ x 10-2
RQ x 10-4
DIKO-12wR-4w 0IU
DIKO-12wR-4w 20IU

Figure S6

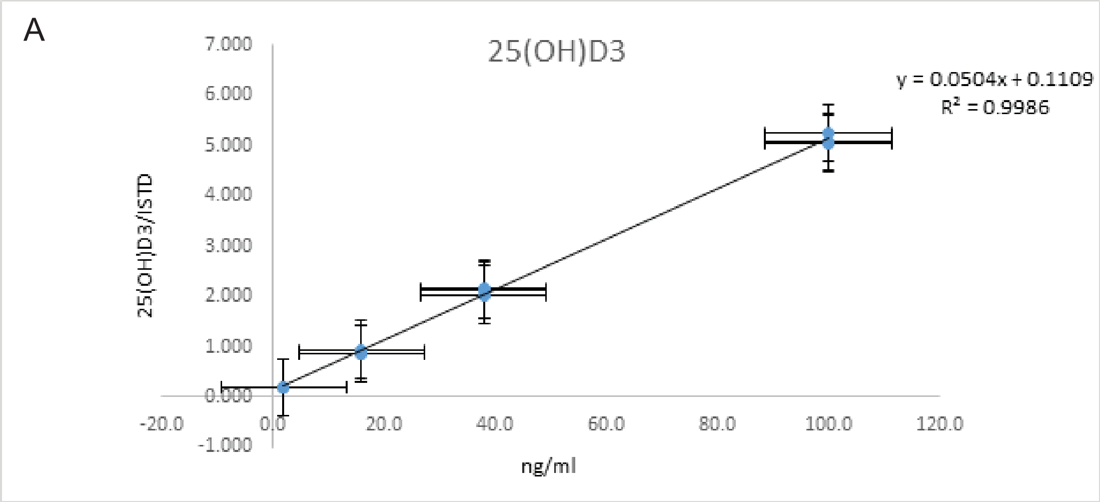
A
25(OH)D3
y = 0.0504x + 0.1109
R² = 0.9986
25(OH)D3/ISTD
7.000
6.000
5.000
4.000
3.000
2.000
1.000
0.000
-1.000
-20.0
0.0
20.0
40.0
60.0
80.0
100.0
120.0
ng/ml

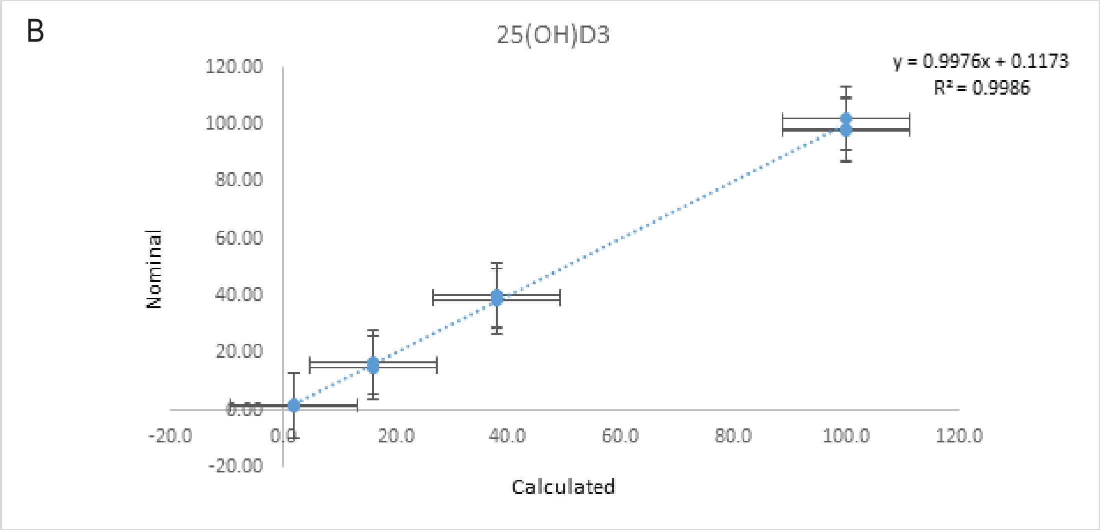
B
25(OH)D3
y = 0.9976x + 0.1173
R² = 0.9986
Nominal
120.00
100.00
80.00
60.00
40.00
20.00
0.00
-20.00
-20.0
0.0
20.0
40.0
60.0
80.0
100.0
120.0
Calculated

Figure S7

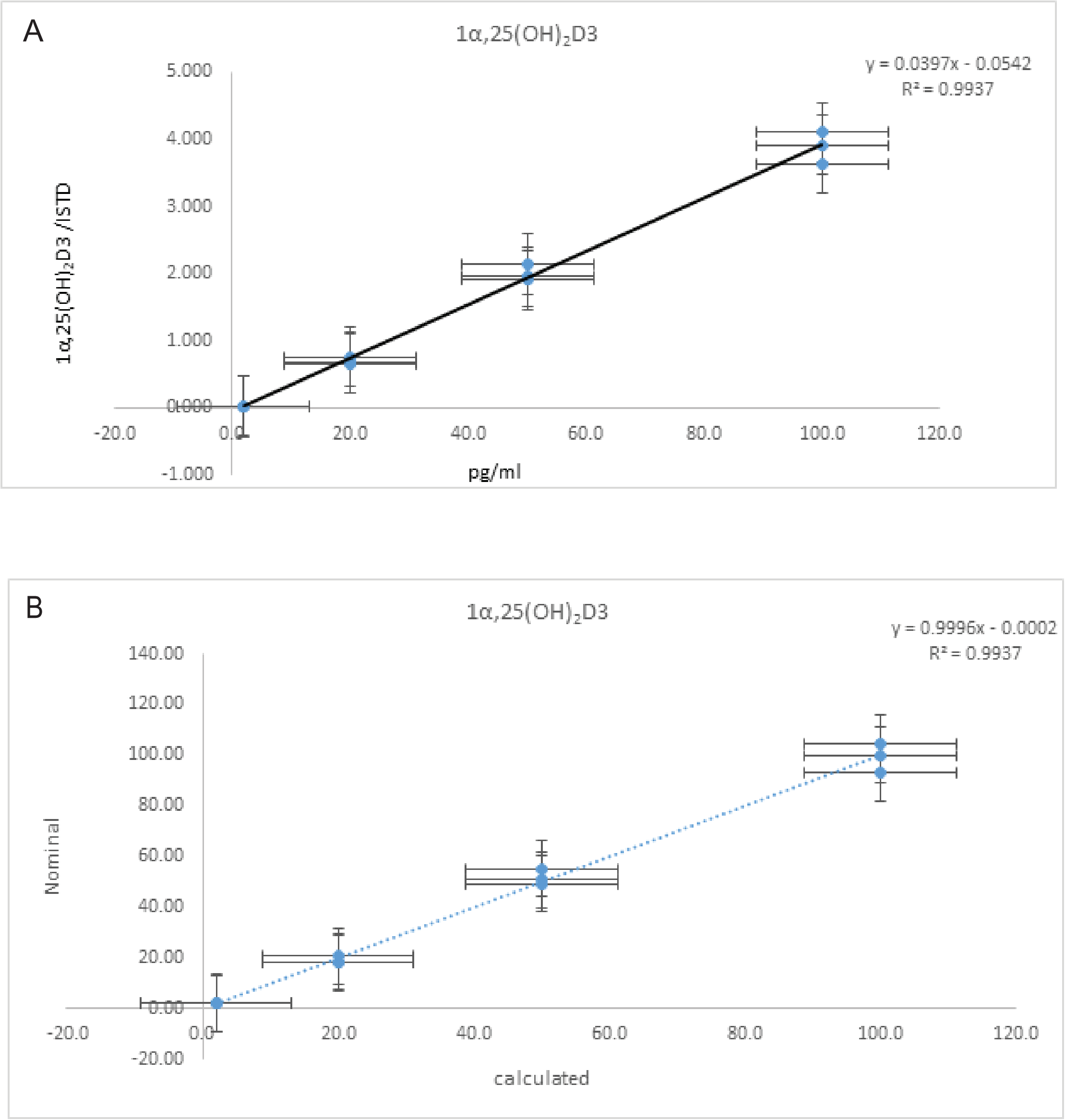

A
1α,25(OH)₂D3
y = 0.0397x - 0.0542
R² = 0.9937
1α,25(OH)₂D3 /ISTD
pg/ml
B
1α,25(OH)₂D3
y = 0.9996x - 0.0002
R² = 0.9937
Nominal
calculated

Figure S8

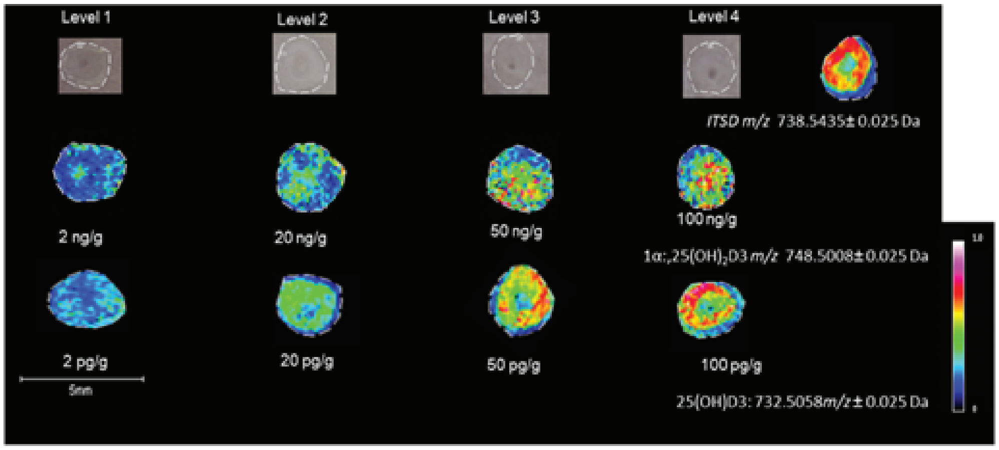
Level 1
Level 2
Level 3
Level 4
ITSD m/z 738.5435±0.025 Da
2 ng/g
20 ng/g
50 ng/g
100 ng/g
1α,25(OH)2D3 m/z 748.5008±0.025 Da
2 pg/g
20 pg/g
50 pg/g
100 pg/g
5mm
25(OH)D3: 732.5058m/z±0.025 Da

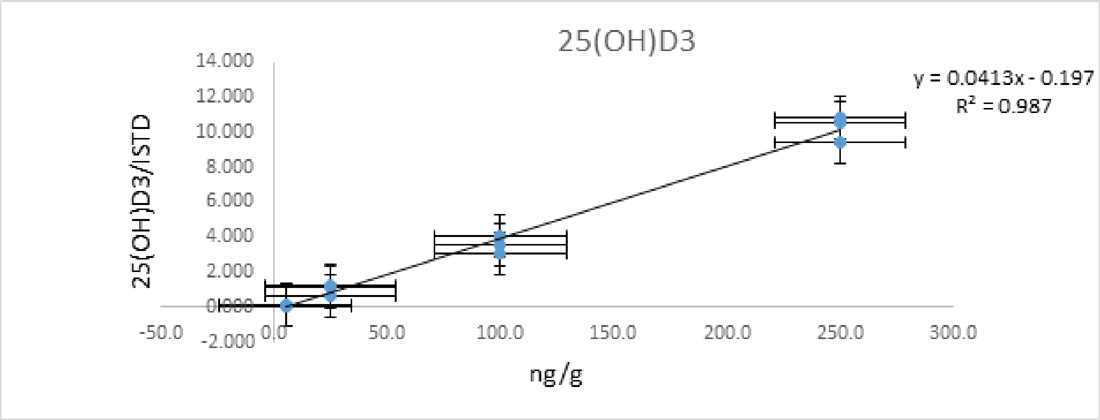
25(OH)D3
y = 0.0413x - 0.197
R² = 0.987
25(OH)D3/ISTD
ng/g

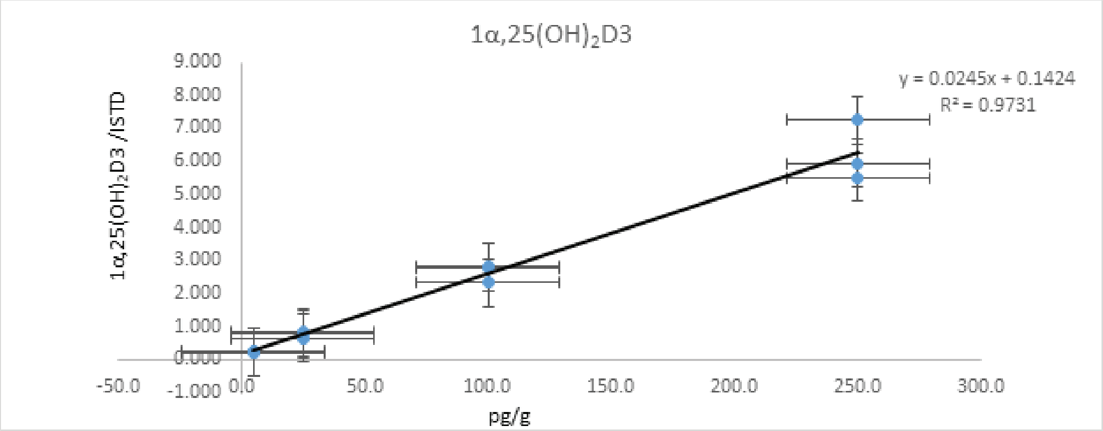
1α,25(OH)2D3
y = 0.0245x + 0.1424
R² = 0.9731
1α25(OH)2D3 /ISTD
pg/g

Figure S9

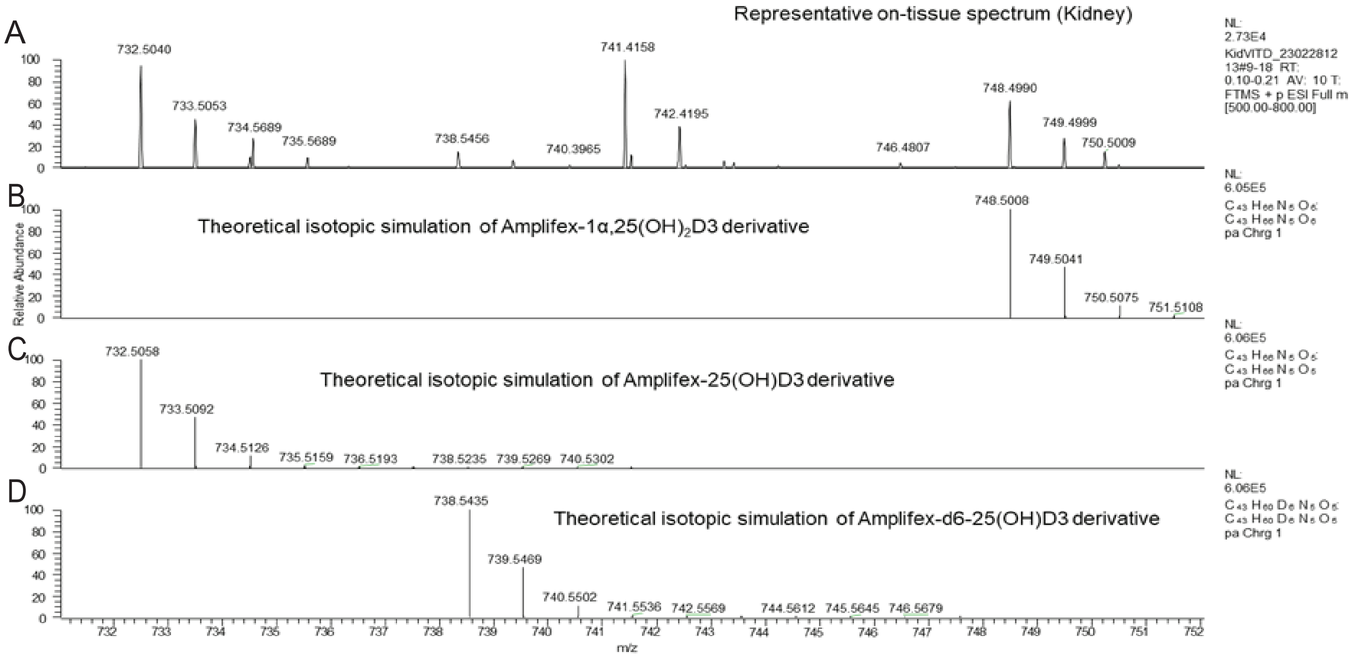

Analyte	Observed protonated mass (Th)	Theoretical protonated mass (Th)	Mass accuracy (ppm)
25(OH)D3	732.5040	732.5058	2.45
1,25(OH)$_2$D3	748.4990	748.5008	2.40

Figure S10

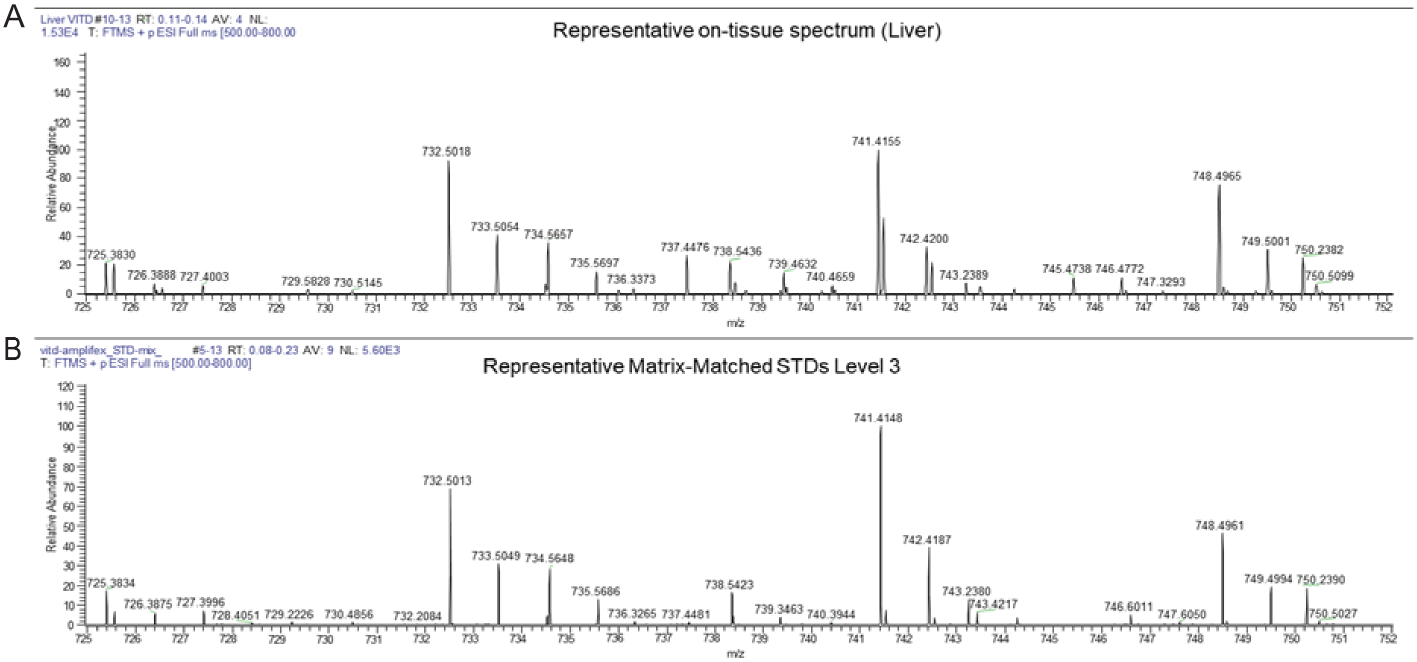

Analyte	On-tissue protonated mass (Th)	Standard mix protonated (Th)	Mass accuracy (ppm)
25(OH)D3	732.5018	732.5013	0.7
1α,25(OH)$_2$D3	748.4965	748.4961	0.5

Figure S11

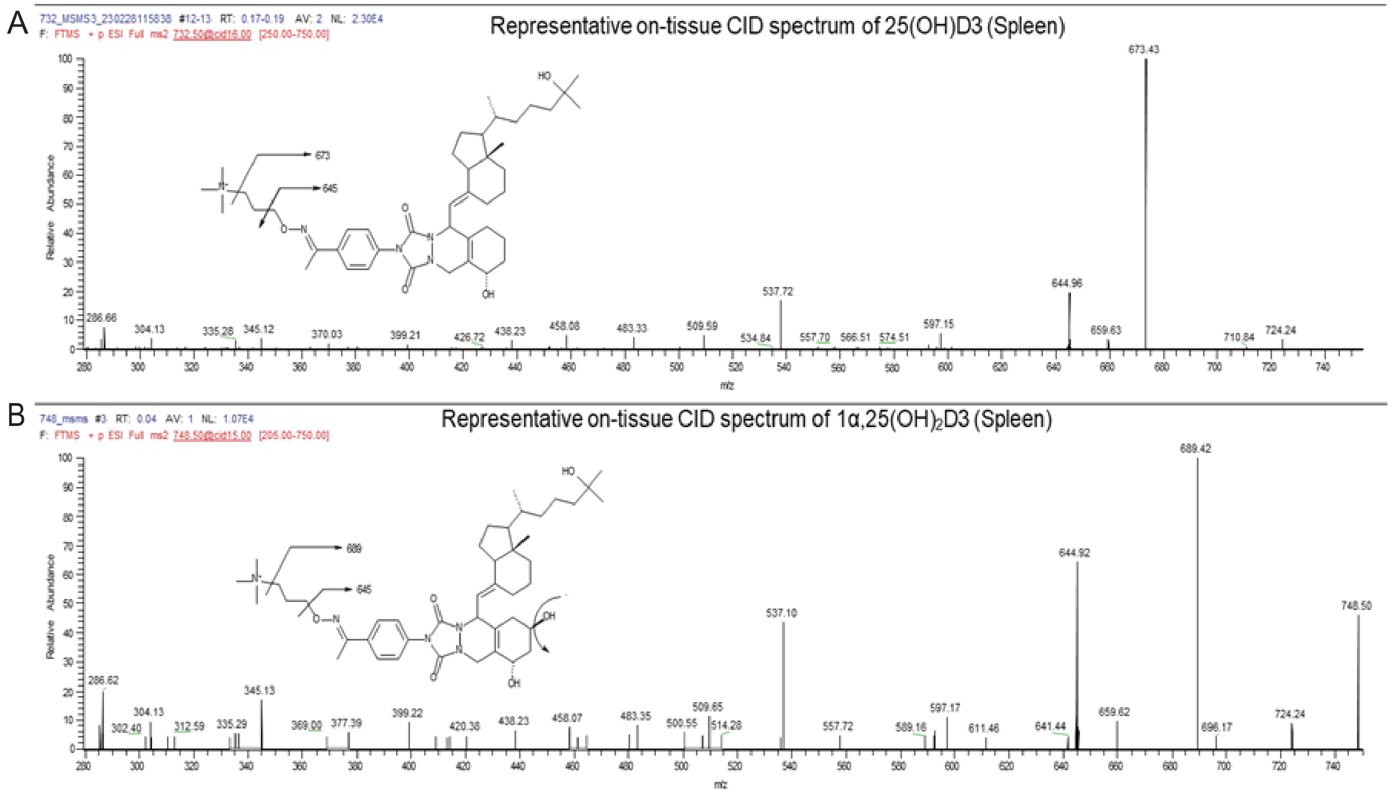
A
732_MSMS3_230228115838 #12-13 RT: 0.17-0.19 AV: 2 NL: 2.30E4
F: FTMS + p ESI Full ms2 732.50@cid16.00 [250.00-750.00]
Representative on-tissue CID spectrum of 25(OH)D3 (Spleen)
Relative Abundance
m/z
673
645
HO
OH
673.43
644.96
537.72
286.66
304.13
335.28
345.12
370.03
399.21
426.72
438.23
458.08
483.33
509.59
534.84
557.70
566.51
574.51
597.15
659.63
710.84
724.24
B
748_msms #3 RT: 0.04 AV: 1 NL: 1.07E4
F: FTMS + p ESI Full ms2 748.50@cid15.00 [205.00-750.00]
Representative on-tissue CID spectrum of 1α,25(OH)2D3 (Spleen)
Relative Abundance
m/z
689
645
HO
OH
689.42
644.92
748.50
537.10
286.62
302.40
304.13
312.59
335.29
345.13
369.00
377.39
399.22
420.38
438.23
458.07
483.35
500.55
509.65
514.28
557.72
589.16
597.17
611.46
641.44
659.62
696.17
724.24

Figure S12

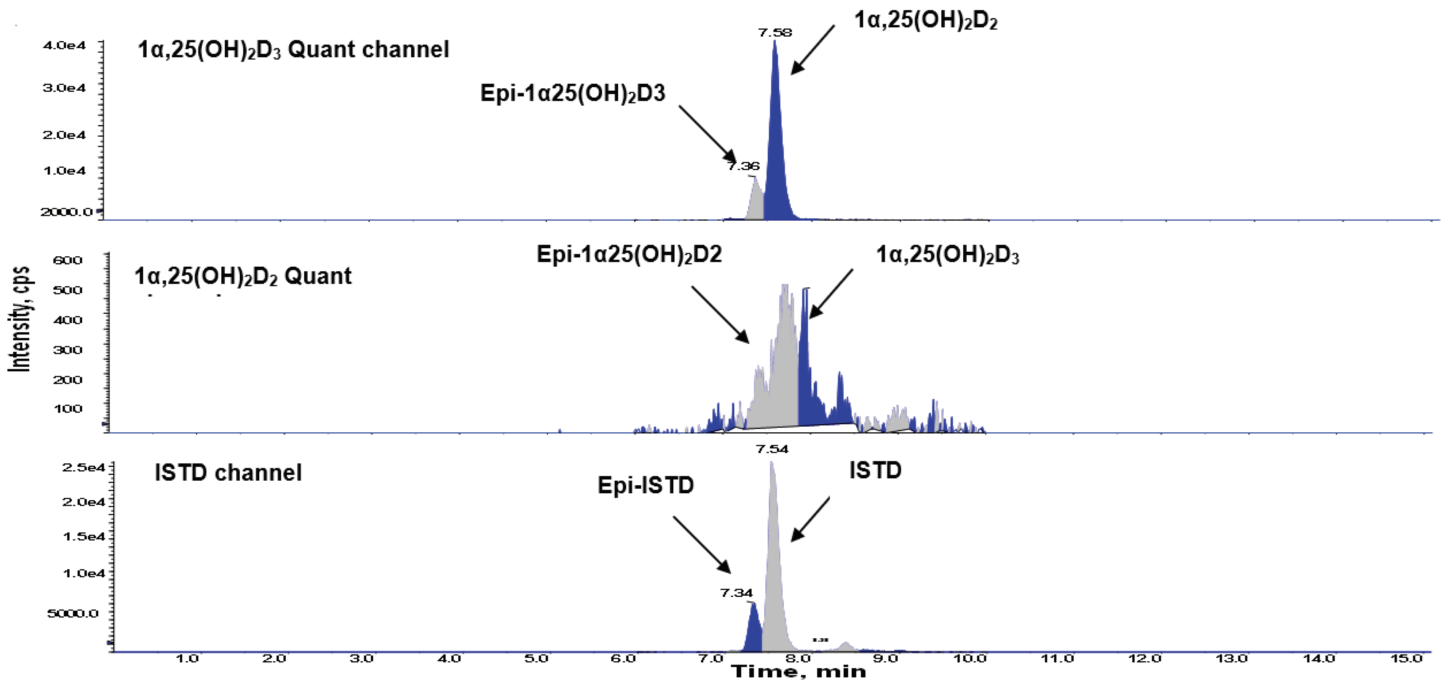

Peak	Component	Retention Time (minutes)
1	ISTD	~7.5
2	1α,25$(OH)_2D_2$	~7.9
3	1α, 25$(OH)_2D_3$	~7.5

Figure S13

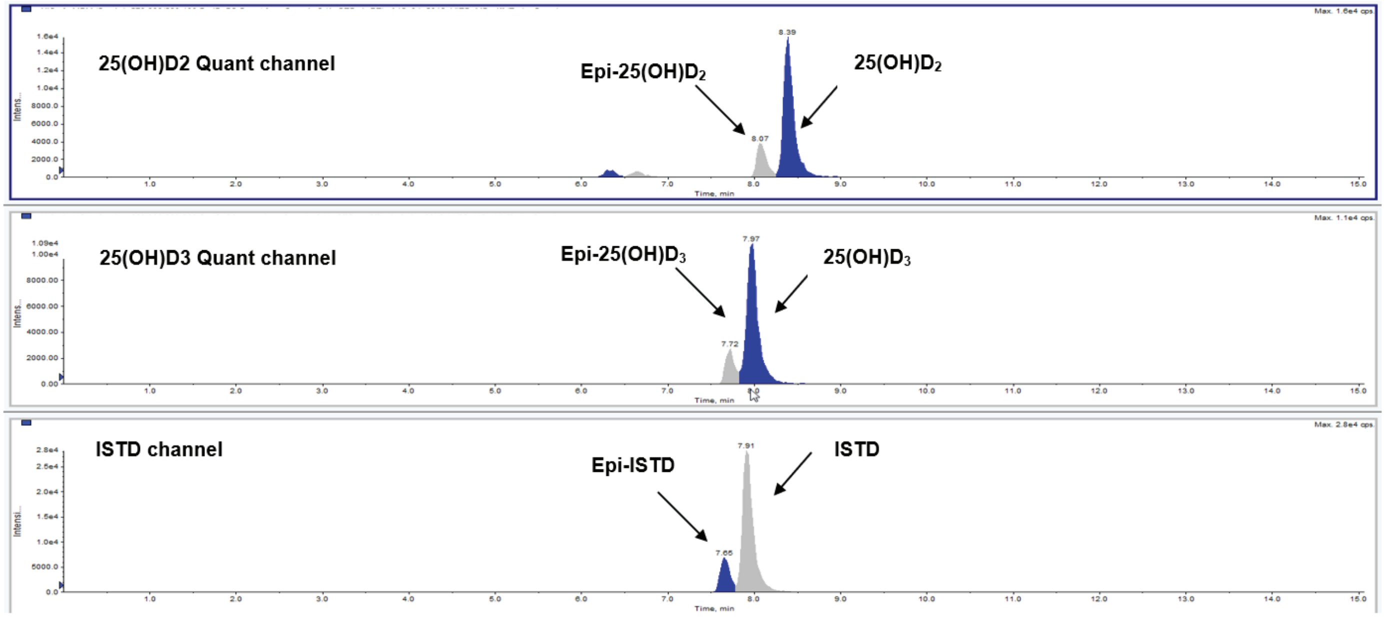

Peak	Component	Retention Time (minutes)
1	ISTD	~7.9
2	25(OH)D_3	~7.9
3	25(OH)D_2	~8.4

Supplemental Figure Legends

Supplemental Figure S1: Tables of plasma LC-MS/MS, MSI relative quantitation, and tissue homogenate LC-MS/MS. A, plasma concentrations of $25(OH)D_3$ (25D, ng/mL) and $1,25(OH)_2D_3$ (1,25D, pg/mL) in the wildtype littermates (WT), *Cyp27b1*-KO (C27KO), M1/M21-DIKO (DIKO), M1/M21-DIKO 12 w rescue diet followed by 4 w of 0 IU vitamin D diet (12wR-4w 0 IU), and M1/M21-DIKO 12 w rescue diet followed by 4 w of 20 IU vitamin D diet (12wR-4w 20 IU). Mouse ID is listed in the ID column. Plasma and tissue samples were taken from the same animals. B, lower limits of quantitation (LLOQ) and lower limits of detection (LLOD) for both plasma and tissue samples for $25(OH)D_3$ (25D) and $1,25(OH)_2D_3$ (1,25D). C, Tissue levels of $25(OH)D_3$ (25D, ng/g) and $1,25(OH)_2D_3$ (1,25D, pg/g) as calculated from relative quantitation of the MSI in kidney, liver, spleen, and thymus for the wildtype littermates (WT), *Cyp27b1*-KO (C27KO), M1/M21-DIKO 12 w rescue diet followed by 4 w of 0 IU vitamin D diet (12wR-4w 0 IU), and M1/M21-DIKO 12 w rescue diet followed by 4 w of 20 IU vitamin D diet (12wR-4w 20 IU). D, confirmatory tissue homogenate levels of $25(OH)D_3$ (25D, ng/g) and $1,25(OH)_2D_3$ (1,25D, pg/g) as calculated from LC-MS/MS in kidney, liver, spleen, and thymus for the *Cyp27b1*-KO (C27KO), M1/M21-DIKO 12 w rescue diet followed by 4 w of 0 IU vitamin D diet (12wR-4w 0 IU), and M1/M21-DIKO 12 w rescue diet followed by 4 w of 20 IU vitamin D diet (12wR-4w 20 IU). Values that fall between the LLOQ and the LLOD are included in the table and highlighted in red.

Supplemental Figure S2: Mass spectrometry imaging for all tissue sections from all mice. Each tissue is displayed with 2 different relative scales to display the metabolite levels appropriately across tissues. Mouse IDs are identical to those found in Fig S1: WT: 101, 102, 103; *Cyp27b1*-KO: 318, 319, 320; M1/M21-DIKO 0 IU: 273, 274, 279; M1/M21-DIKO 20 IU: 296, 297, 302. Intensity was normalized by stable isotope internal standard protonated mass. Signal intensity is depicted by color on the scale shown.

Supplemental Figure S3: Serum profiles for experimental mice. Serum was collected and measured for calcium (A) and phosphate (B) (n=6, male/female combined). Weights of mice (C) were examined as male and female separately. EDTA-treated plasma was collected and assayed for PTH (D) and intact FGF23 (iFGF23, E) (n=6, male/female combined). Bone mineral density was measured and femoral BMD (F) was displayed for male and female separately. One-way ANOVA with tukey post-test: ****, $p<0.0001$; ***, $p<0.001$; *, $p<0.05$.

Supplemental Figure S4: Bland-altman analysis comparing the two methods of MSI vs. Tissue Homogenate LC-MS/MS. Ratio of (MSI / Tissue Homogenate) vs. Average is shown for kidney, liver, spleen for $25(OH)D_3$ (A) and liver, spleen, and thymus for $1,25(OH)_2D_3$.

Supplemental Figure S5: Gene expression for megalin (*Lrp2*) and cubulin (*Cubn*) was performed in the kidney, liver, spleen, and thymus for mice on the M1/M21-DIKO 12 w rescue diet followed by 4 w of 0 IU vitamin D diet (12wR-4w 0 IU), and M1/M21-DIKO 12 w rescue diet followed by 4 w of 20 IU vitamin D diet (12wR-4w 20 IU). A, data from the M1/M21-DIKO (12wR-4w 0 IU) plotted on same axis. Split axis to show expression differences between tissues. One-way ANOVA with tukey post-test: ****, $p<0.0001$; ***, $p<0.001$; *, $p<0.05$. B, data displayed from both the 0 IU and 20 IU diets. Unpaired t-tests were performed: ****, $p<0.0001$; ***, $p<0.001$; *, $p<0.05$ - 20 IU vs 0 IU.

Supplemental Figure S6: a) Calibration curve of $25(OH)D_3$ dynamic range (2-100 ng/ml) n=3 per time point. **b**) Nominal Vs Back calculated values. Errors bars in standard error. Calculation was performed using Excel for Office 365.

Supplemental Figure S7: a) Calibration curve of $1\alpha,25(OH)_2D_3$ dynamic range (2-100 pg/ml) n=3 per time point. **b**) Nominal Vs Back calculated values. Errors bars in standard error. Calculation was performed using Excel for Office 365.

Supplemental Figure S8: Matrix-matched standard-Tissue mimetic model standards curves. Intensity was normalized by stable isotope internal standard protonated mass. Signal intensity is depicted by color on the scale shown.

Supplemental Figure S9: Representative FTMS spectrum of **a)** Kidney tissue section along with theoretical monoisotopic simulation of **b)** 1α,25$(OH)_2D_3$ **c)** 25$(OH)D_3$ and **d)** ISTD of d6-25$(OH)D_3$ as Ampiflex derivatives with mass accuracy calculation.

Supplemental Figure S10: Representative FTMS spectrum of **a)** Liver tissue section **b)** Matrix-matched standard level along with mass accuracy calculation.

Supplemental Figure S11: Representative collision induced dissociation (CID) spectra of spleen tissue of **a)** 25$(OH)D_3$ and **b)** 1α,25$(OH)_2D_3$ along with corresponding embedded proposed fragmentation pattern.

Supplemental Figure S12: Representative MRM chromatogram of 1α,25$(OH)_2$D metabolites as Ampiflex® derivatives in target tissues.

Supplemental Figure S13: Representative MRM chromatogram of 25(OH)D metabolites in target tissues.

Supplemental Methods and Tables

Supplemental Table 1: TaqMan gene expression primers

**Gene**	**Catalog #**	**Assay ID**	**Dye**
Gapdh	4352339E	Mm99999915_g1	VIC
Cyp24a1	4331182	Mm00487244_m1	FAM
Cyp27b1	4331182	Mm01165918_g1	FAM
Vdr	4331182	Mm00437297_m1	FAM
Il4	4331182	Mm00445259_m1	FAM
Il10	4331182	Mm00439614_m1	FAM
Il17a	4331182	Mm00439618_m1	FAM
Il1b	4331182	Mm00434228_m1	FAM
Il2	4331182	Mm00434256_m1	FAM
Il6	4453320	Mm00446190_m1	FAM
Tnfa	4331182	Mm00443258_m1	FAM

Lrp2	4331182	Mm01328171_m1	FAM
Cubn	4331182	Mm01325040_m1	FAM

Analysis of 25(OH)D₃ and 1α,25 (OH)₂D₃ in plasma and tissue homogenate: Vitamin D metabolites 25(OH)D_3 and 1α,25(OH)$_2$$D_3$ were analyzed by Liquid Chromatography Tandem Mass Spectrometry (LC-MS/MS). An automated Multipurpose Sampler (MPS) equipped with a pre-column micro solid phase extraction (ISTP hardware Kit, Anatune, CA, UK) was used for biomatrix cleaning and analyte extraction (Gerstel, GmBH). Pre-column derivatization only for 1α,25(OH)$_2$$D_3$ was performed using Ampiflex® (Sciex, Chemistry and Consumables R&D, Framingham, MA, US). The method was adapted from Higashi et al., 2010 and Shan Xu et al., 2022 (1, 2).

Preparation of Standards and Quality Control Samples: For 1α,25(OH)$_2$$D_3$: Stock solution of both 1α,25(OH)$_2$$D_3$ and d6-1α,25(OH)$_2$$D_3$ (as internal standard (ISTD)) (100µg/ml, in methanol) were used to preparate working solutions, standards calibration curves and quality controls (QCs). 1α,25(OH)$_2$$D_3$ stock solution was further diluted with surrogate biomatrix (Golden Mass Spect Gold® Human Serum, Ultra-Low Vitamin D, Lipid Free, Cell Culture Collective INC, CA, US) to provide calibration standards in the range of 5-100pg/ml and an ISTD concentration of 100 pg/ml. Quality control samples were independently prepared in the surrogate biomatrix at four different concentrations (2,20,50,100 pg/m)l corresponding to (LLOQ, QCL, QCM, QCH). For 25(OH)D_3, commercially available lyophilized four-point calibration standards and QC material (2, 16, 38 and 100ng/ml) corresponding to (LLOQ, QCL, QCM and QCH) respectively were prepared according to manufacture specifications (Chromsystem, München, GmBH). Stock solution of d6-25(OH)D_3 at 100ug/ml in methanol was prepared and used as ISTD for quantitation. Quality surrogate calibration standards were prepared freshly daily from the working solutions. All stock and working solution were stored at -80ºC until analysis.

Sample extraction plasma: 125 µl of plasma sample was placed in a standard 2 ml glass screw top autosampler vial and the vial capped using a magnetically transportable PolyMag™ cap (GERSTEL, Germany). The sample is then placed on the vial tray of the MultiPurpose Sampler (MPS). 50 µL of internal standard solution (d6-25OH-D_3, 100ng/ml)) and 50µL (1d6-1α,25(OH)$_2$D3, 100 pg/ml) were added to the sample leading to a final concentration of 25 ng/ml) and 25 pg/ml respectively, followed by 200 µL of a 0.2 M zinc sulphate solution to enhance the sensitivity of the assay. Following this, 500 µl of methanol is added to the vial to precipitate the proteins. The vial was then moved using magnetic transport to the CF-100 centrifuge whereby the contents were thoroughly vortexed for 1 minute to assist in the protein precipitation. The vial was then centrifuged at 3000 rpm for 1 minute to separate the proteins from the supernatant. A 10 mg C18 Vitamin D ITSP SPE cartridge was solvated with 100 µl of methanol and then equilibrated with 100 µL of HPLC grade water. 500 µL of the supernatant is then loaded onto the SPE cartridge, before the cartridge was washed with 100 µL of 70 % methanol in water. The cartridge was then dried with 250 µL of air. Analytes are eluted with one 250 µL aliquot of methanol into a 500 µl high recovery vial. The polarity of the final solution is then adjusted by the addition of 100 µL of HPLC grade water, to improve the peak shape of the analytes. Samples were then divided in two aliquots of 150µL. One aliquot was transferred to a 200µL insert (2ml amber vial) for LC-MS/MS analysis. The other 150µL was dried under gently stream of Nitrogen at RT and then subjected to a one-step derivatization using Ampiflex® Diene as reagent to improve the ionization efficiency. To the dried residue, 30µL of Ampiflex solution was added as per manufacture specification, vortexed 30 and incubated for 60 min at RT. Then, sodium Borohydride solution (10mM) (10µL) was then added to reduce the complexity of the Chromatogram by reducing the enamine double bond formation of the Ampiflex derivative. Finally, MiliQ water (30µL) was added to the sample, vortexed for 15 s, and then transferred into 200µL HPLC inserts (2ml amber vials) for further LC-MS/MS.

Sample extraction for tissue homogenate: Tissue sections (~50 mg) were homogenized by adding 700µL of hexane/acetone mixture (1:1, v/v) and using ultrasonication for 1min, amplitude 80% and interval of 0.5-0.9s (UP50H, MMTG, US) in a 1.2 mL Eppendorf and then added 50µL and 100µL of 25(OH)D_3 (100ng/ml) and 1α,25$(OH)_2D_3$ (100pg/ml) respectively. Samples were kept in ice bath during homogenization. 300µLof MiliQ water was added to the mixture, vortexed for 30s, ultrasonicated for 10 min and centrifuged for 10 min at 15,000 RPM. The supernatant (~600µL) was dried down by evaporation using a gently steam of nitrogen at RT. Samples were reconstituted in (80:20 (v/v)) water: methanol and transferred to the MPS (200µL) and then followed sample preparation steps are per plasma analysis previously described in Experimental Procedures except for the addition of ISTDs.

***Liquid Chromatography Tandem Mass Spectrometry (LC-MS/MS) Conditions*:** Standards and samples were separated using a Zorbax-extended C18 (100x2.1mmx3.0µm) HPLC column (Agilent, CA, US) on a Nexera UPLC system (Shimadzu, Kyoto, Japan).The chromatography mobile phases for 25(OH)D3 consisted of 0.1% (v/v) formic acid in water: methanol (50:50, v/v) (Eluent A) and 0.1% (v/v) formic acid in methanol (Eluent B). The chromatographic conditions for 1α,25$(OH)_2$D3 consisted of 0.1% (v/v) formic acid in water (Eluent C) and 0.1% (v/v) formic acid in water: methanol (70:30, v/v) (Eluent D). A gradient elution was performed as per **Table S2**. Same gradient elution was used for both analytes.

Table S2: HPLC Gradient Profile

	**25(OH)D_3**		**1α,25$(OH)_2D_3$**	
Time	A (%)	B (%)	C (%)	D(%)
0.00	100	0	100	0
3.00	100	0	100	0
10.00	0	100	0	100
10.10	100	0	100	0
15.10	100	0	100	0

The HPLC column flow rate was set to 0.35 ml/mi throughout the chromatographic run and the column temperature was maintained at 40°C. A divert valve was used to minimize the contamination of the MS system and it was set to waste in the time interval of 0-5 min and 11-15 min.

Mass Spectrometry analysis (Qtrap Ready 5500+ Mass Spectrometer (MS), SCIEX, Framingham, US) was caried out in positive ion mode using the TurboIonSpray Electrospray ionization (ESI) for detection of 1α,25$(OH)_2D_3$ and the corresponding ISTD and Atmospheric pressure Chemical Ionization (APCI) for detection of 25(OH)D_3 and the corresponding ISTD. Multiple Reaction Monitoring (MRM) was used as acquisition mode. MS source parameters were set as follows: CAD: (Arb) 6, CUR (Arb): 40, I Spray Voltage (V): 5000, GSI (Arb): 35, GSII (Arb): 40, TEM (°C): 450 and corona current (APCI): (5µA). For MRM transitions, please see Table S2. 25(OH)D_2 and 1α,25$(OH)_2D_2$ were also monitored (not quantified) for information only.

Table S3: Multiple Reaction Monitoring Table

**Compound**	**Parent (m/z)**	**Quantifier (m/z)**	**Qualifier (m/z)**	**CE (V)**	**DP (V)**	**CXP (V)**	**EP (V)**
25(OH)D_3	401.3	383.3	159.1	23/47	91	10/16	10
25(OH)D_2	413.3	395.3	159.1	23/49	96	10/12	10
d6-25(OH)D_3	407.3	389.3	N/A	23	101	10	10
1α,25$(OH)_2D_3$	748.5	689.2	644.8	21/35	99	9/10	10

1α,$25(OH)_2D_2$	761.5	701.2	644.9	17/29	115	10/11	10
d6-1α,$25(OH)_2D_3$	754.5	695.4	N/A	16	96	10	10

N/A: Not applicable, CE: Collision energy, DP: Delustering potential, CXP: Collision cell exit potential and EP: Exit potential.

Acquisition and validation parameters: Duplicate injections were made of each sample preparation and analysis reposted as the average (ng/ml) for plasma and (ng/g) for tissues for $25(OH)D_3$ and (pg/ml) and (pg/g) for plasma and tissue respectively for 1α,$25(OH)_2D_3$. For each analyte (native and derivatized) calibration curves were plotted with peak area ratios of vit D metabolites to the respective ISTD versus a range of analyte concentration in the corresponding units. For both analytes, two peak were observed (corresponded to their diastereomers) and summed areas were used for quantitation.

For $25(OH)D_3$, dynamic range (triplicate injection) was from 2-100 ng/ml (plasma) equivalent to 5-250 ng/g (tissue homogenate) with a lower Limit of Quantitation (LLOQ) of 2ng/ml (plasma) and 5 ng/g (tissues). The calibration curve for plasma was used for quantitation of tissue homogenate factoring a dilution factor of 1.5 in the calculations. Three replicates for each concentration level (2,16,38 and 100 ng/ml) were processed. The average percentage of Coefficient of Variation (CV%) was 9.5 for calibrators and 8-13% for QCs with a mass accuracy of between 77 (at LLOQ) -106% for both calibration and QCs (**Fig. S5**).

For 1α,$25(OH)_2D_3$, dynamic range (triplicate injection) was form 2-100pg/ml (plasma) equivalent to 5-250 ng/g (tissue homogenate) with a lower Limit of Quantitation (LLOQ) of 2pg/ml (plasma) and 5 pg/g (tissues). The calibration curve for plasma was used for quantitation of tissue homogenate factoring a dilution factor of 2.5 in the calculations. Three replicates for each concentration level (2, 20, 50 and 100 ng/ml) were processed. The average percentage of Coefficient of Variation (CV%) was 5.7 for calibrators and 10-17% for QCs with a mass accuracy of between 89 -104% for both calibration and QCs (**Fig. S6**).

Accuracy and precision replicated samples at two concentration levels were analyzed in separate runs to determine intra-day precision and accuracy. Intra-day accuracy and precision were calculated by processing six replicates at two concentration levels. Precision of the assay was estimated by the CV% for each concentration level. Accuracy was represented as recovery% from the nominal concentration as per **Table S4.**

Table S4: Tissue homogenate accuracy and precision result summary

$25(OH)D_3$ Level 2: nominal (ng/ml)	Calculated concentration (ng/L)	Recovery (%)	1α,$25(OH)_2D_3$ Level 2 nominal (pg/ml)	Calculated concentration (pg/L)	Recovery (%)
16.8	16.1	95.8	20.2	21.2	104.9
	16.6	99.0		18.8	93.1
	15.8	94.0		19.6	97.2
	15.3	90.8		20.7	102.7
	16.9	100.4		18.7	92.5
	16.1	95.9		18.7	92.5
**CV (%)**	**3.3**			**4.5**	
$25(OH)D_3$	Calculated concentration	Recovery (%)	1α,$25(OH)_2D_3$ Level 4	Calculated concentration	

Level 4: nominal (ng/ml)	(ng/L)		nominal (pg/ml)	(pg/L)	Recovery (%)
100.1	95.7	95.6	100.3	100.1	99.8
	105.2	105.1		103.9	103.6
	112.2	112.1		98.1	97.8
	114.2	114.1		108.4	108.0
	107.8	107.7		113.0	112.7
	111.6	111.50		89.5	89.2
**CV (%)**	**5.7**			**7.4**	

Mass Spectrometry Imaging: Matrix-matched standard-Tissue mimetic model: The protocol was adapted from Groseclose et al., 2018 (3). The process is outlined in **Fig. S9** which is adapted from the aforementioned protocol. For a given model, approximately 2 g (~250 mg/layer × eight layers) of the concordant Vit D metabolites free (Golden West Biological, CA, USA) bulk tissue was homogenized without additional solvent using the FastPrep 24 bead homogenizer (MP Biomedicals) and stainless-steel lysing matrix (MP Biomedicals). The bulk homogenate was then aliquoted into four homogenizing vials using a positive displacement pipette. After determining the mass of the tissue homogenate in each vial, an appropriate amount of the standard was spiked into each homogenate to yield the desired final tissue concentration for (5-250 ng/g) for $25OHD_3$ and (5-250 pg/g) for $1\alpha,25(OH)_2D_3$. The volume of standard spiked into each homogenate was maintained below approximately 3% (w/w) to minimize the impact on the native tissue density. A mold was prepared from a 3 ml syringe by drawing back the plunger and removing the luered end. Enough of the blank tissue homogenate was added to the mold to sufficiently coat the plunger of the syringe (~250 µL). The mold was immersed into dry ice cooled isopropanol to freeze the homogenate layer without submerging the open end of the syringe. Once completely frozen, the tissue plug was removed from the mold by carefully depressing the syringe plunger. A longitudinal (vertical) cross-section of the tissue plug sampling all concentration levels was then obtained by cryosectioning as previously described for target tissue sections. Sections were collected at the same thickness as the tissue to be quantified and thaw-mounted to the same substrate (indium tin oxide (ITO) coated microscope slide) as the sample to be quantified so that both exhibited to the same sample preparation conditions.

Histological Staining: Tissue sections (kidney and spleen) were stained using hematoxylin (0.02 g/L in ethanol) (Sigma-Aldrich, Dorset, UK) and eosin (0.003g/L in water + 1% Na_2CO_3) dyes as follows: fixed in 20°C acetone (Honeywell, UK) for 10 min and air-dried. Sections were rehydrated in 70 % v/v ethanol (EtOH) (≥99.8 %, Honeywell, Arlington, UK) (2 min) and tap water (5 min). They were then immersed in hematoxylin dye (6 min), rinsed in water (2 min) and placed in a solution of 10 % v/v acetic acid (Sigma Aldrich, Dorset, UK) in 95 % EtOH (1 min). To enhance the efficacy of the hematoxylin stain, slides were rinsed in water for 15 min in a bluing step. Sections were transferred to eosin dye for 15 s and dipped in water 2-3 times rapidly or until streaking stopped. Stained sections were dehydrated in increasing concentrations of EtOH (50 % - 100 % v/v) for 2 min each and cleared by two changes of xylene (reagent grade, Fisher Scientific, Loughborough, UK) also at 2 min each. Histological mount (Histamount National Diagnostics, Atlanta, US) was applied before sections were completely dry and a glass coverslip was applied. A 600-dpi image was taken of tissues on slides using a scanner (Epson Perfection V330, software version3.9.2.5 EN, Seiko Epson Corporation, Nagano, Japan).

Supplemental References

1. Higashi, T., Shimada, K., andToyo'oka, T. (2010) Advances in determination of vitamin D related

compounds in biological samples using liquid chromatography-mass spectrometry: a review J Chromatogr B Analyt Technol Biomed Life Sci **878**, 1654-1661 10.1016/j.jchromb.2009.11.026

2. Xu, S., Ni, R., Lv, L., Chen, R., Chen, Y., Huang, F. *et al.* (2022) Simultaneous determination of vitamin D metabolites 25(OH)D J Mass Spectrom Adv Clin Lab **24**, 65-79 10.1016/j.jmsacl.2022.04.001
3. Barry, J., Barry, J. A., Groseclose, M. R., Fraser, D. D., andCastellino, S. (2018) Revised Preparation of a Mimetic Tissue Model for Quantitative Imaging Mass Spectrometry Protocol Exchange 10.1038/protex.2018.104
